# Rebound of self-lubricating compound drops

**DOI:** 10.1126/sciadv.aay3499

**Published:** 2020-03-13

**Authors:** Nathan Blanken, Muhammad Saeed Saleem, Carlo Antonini, Marie-Jean Thoraval

**Affiliations:** 1 State Key Laboratory for Strength and Vibration of Mechanical Structures, Shaanxi Key Laboratory of Environment and Control for Flight Vehicle, International Center for Applied Mechanics, School of Aerospace, Xi’an Jiaotong University, Xi’an 710049, P. R. China.; 2 Department of Materials Science, University of Milano-Bicocca, Milan, Italy.; 3 Cellulose and Wood Materials, Swiss Federal Laboratories for Materials Science and Technology (Empa), Dübendorf, Switzerland.

## Abstract

Drop impact on solid surfaces is encountered in numerous natural and technological processes. Although the impact of single-phase drops has been widely explored, the impact of compound drops has received little attention. Here, we demonstrate a self-lubrication mechanism for water-in-oil compound drops impacting on a solid surface. Unexpectedly, the core water drop rebounds from the surface below a threshold impact velocity, irrespective of the substrate wettability. This is interpreted as the result of lubrication from the oil shell that prevents contact between the water core and the solid surface. We combine side and bottom view high-speed imaging to demonstrate the correlation between the water core rebound and the oil layer stability. A theoretical model is developed to explain the observed effect of compound drop geometry. This work sets the ground for precise complex drop deposition, with a strong impact on two- and three-dimensional printing technologies and liquid separation.

## Teaser

An oil shell encapsulating a water drop promotes rebound after impact on a solid surface, irrespective of substrate wettability.

## INTRODUCTION

The impact of a drop on a solid or liquid surface is encountered in a wide range of applications, including combustion, three-dimensional (3D) printing, biological microarrays, pharmaceutics, and food industry (*1*–*3*). Many technologies, such as steel strip manufacturing (*4*), combustion (*5*), and agricultural sprays (*6*), use emulsion droplets of immiscible liquids. Using an emulsion as the impacting drop (*4*, *6*, *7*), fluid rheology can be modified (*8*–*10*), or splashing sheets can locally be broken up (*11*). With the emergence of additive manufacturing technologies (*3*, *12*, *13*), macroscopic compound drops can be used in a wide range of practical applications. One of the main challenges for these applications is to control the deposition process of the impacting drop. Knowledge of the spreading, splashing, and rebound behavior (*14*) of compound drops is therefore of crucial importance.

Only a few studies have looked at the impact of compound drops on a solid surface (*15*–*20*) or a liquid pool (*21*–*25*). Partial rebound of the impacting liquids after impact on a solid surface was observed by Chen *et al.* (*15*) and Liu and Tran (*18*), but they did not explain the underlying mechanism. Complete rebound of the core and shell liquids was observed on a hot surface (*26*, *27*) due to the Leidenfrost effect (*28*–*32*).

Here, we show that rebound of a water-in-oil compound drop on a solid surface is due to the lubrication of the solid surface by the oil shell of the compound drop itself, preventing direct contact between the water core and the solid surface. We refer to this mechanism as self-lubrication. In this study, we systematically investigated the parameters affecting rebound of the water core, including impact speed, water volume fraction, compound drop geometry, and substrate wettability. Above a critical impact velocity, core rebound is absent. We demonstrate that the suppression of core rebound at high impact speed is caused by the breakup of the lubricating oil layer between the water core and the solid surface, and we propose a model to predict the critical impact velocity above which this occurs. The self-lubrication mechanism is similar to rebound due to the cushioning effect of an air film (*33*, *34*), a vapor layer (*28*–*31*, *35*), or the liquid film on lubricated surfaces (*36*). However, in the case of compound drops, the lubrication layer is provided by the impacting drop itself, and rebound is therefore independent of the wetting properties of the solid surface. On the one hand, knowledge of the rebound conditions can help mitigate rebound in deposition applications. On the other hand, rebound of the core of a compound drop could be beneficial by providing a means of mechanical separation of immiscible liquids.

## RESULTS

### Impact of a compound drop

The compound drops used in this case study were millimetric drops consisting of a water core inside a 5-cSt silicone oil shell. These compound drops were accelerated by gravity before impact on a target substrate. Two different drop-generation methods were used: (i) the coaxial needle method (*37*) and (ii) the injection method. We will first present results obtained using a coaxial needle. These results provide a phenomenological overview of the compound drop impact event. As a second step, we will present and discuss results obtained using the injection method to highlight the effect of the drop-generation method on compound drop geometry and, consequently, on the impact outcome.

For the coaxial needle method, compound drops were produced by infusing silicone oil through the outer needle [outer diameter (OD) 0.81 mm] of a coaxial needle. Simultaneously, deionized water was infused through the inner needle (0.23 mm OD). By continuous infusion of the two liquids, water-in-oil drops with a compound diameter of *D*_o_ = 2.3 to 2.4 mm were produced at regular time intervals. Impacting drops were further characterized by their water volume fractions relative to the total volume α = Ω_w_/Ω_0_ and impact heights *h* (defined as the distance between the needle tip and the substrate) or, alternatively, by the corresponding impact velocities *V*. The corresponding Weber number was defined as *We* = [αρ_w_ + (1 − α)ρ_o_]*D*_o_*V*^2^/σ_o_, where ρ_w_ = 998 kg/m^3^ and ρ_o_ = 913 kg/m^3^ are the densities of water and oil, respectively, and σ_o_ = 20 mN/m is the surface tension of the oil. The Weber number was defined such that it is proportional to the ratio of the total kinetic energy and the surface energy of the outer surface of the compound drop. Since the difference between ρ_w_ and ρ_o_ is small, *We* only depends slightly on α. Compound drops impacted onto horizontal, hydrophilic glass substrates (static contact angle of <5° for both water and oil). The interfacial tension σ_ow_ of the water-oil interface was experimentally determined to be 42 mN/m. Since σ_o_ + σ_ow_ < σ_w_, the water core remains wetted by oil throughout an impact event.

**Fig. 1:**
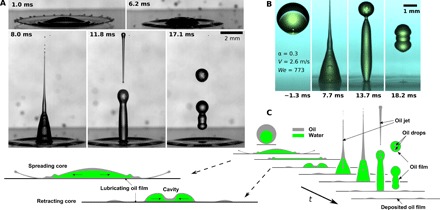
Impact of a compound drop of water with volume fraction α *=* 0.3 on a hydrophilic surface. (**A**) Black and white image sequence of an impact from an impact height of *h* = 0.33 m (impact velocity *V* = 2.4 m/s, *We* = 659). (**B**) Color image sequence for *h* = 0.39 m (*We* = 772). The water was dyed through addition of a fluorescein salt to distinguish it from the oil. Images were postprocessed to remove spots due to dust on the sensor. (**C**) Schematic vertical cross sections of an impacting compound drop, illustrating horizontal splashing of oil, the formation and collapse of a cavity, the ejection of a vertical oil jet, the entrapment of oil in the core, and rebound of the core.

Figure 1 illustrates the image sequence of a characteristic drop impact event [α = 0.3; *We* = 659 in black and white (Fig. 1A) and *We* = 772 in color image using water with fluorescent dye (Fig. 1B)]. Multiple phenomena come into play before rebound of the water core is lastly achieved. In the first few milliseconds after impact, the compound drop starts spreading. The low–surface tension silicone oil shell experiences the well-known corona splash, in which the lamella lifts off (*1*, *38*) because of aerodynamic interaction with the surrounding air and breaks up at the rim, causing micrometric-sized drop ejection (Fig. 1A, 1.0 ms). The characteristic Weber number associated with the core, *We*_w_ = ρ_w_*D*_w_*V*^2^/σ_ow_, where *D*_w_ = α^1/3^*D*_o_ is the diameter of the core, is substantially lower than the Weber number of the entire drop. Therefore, no water splash is observed. Water spreads on top of the spreading lubricating oil layer. After the inertia-driven spreading of the liquids, the oil keeps wetting the glass, due to the low receding contact angle of the oil on the hydrophilic glass surface. The deposited lubricating oil layer prevents contact between the hydrophilic substrate and the water core, allowing the water core to recoil through a self-lubrication mechanism, i.e., lubrication induced by the compound drop itself, particularly by the oil shell surrounding the water.

Upon contraction of the water core rim, a cavity forms in which oil from the shell accumulates (Fig. 1A, 6.2 ms). The collapse of the cavity about the vertical axis leads to the ejection of a high-velocity, vertical oil jet. The jet breaks up into micrometric drops achieving velocities over 10 times the impact velocity (Fig. 1A, 8.0 ms). Moreover, the collapse of the oil-filled cavity causes the entrapment of small oil drops, resulting in a double emulsion. Similar jets have also been observed with pure water drops impacting on hydrophobic surfaces (*39*). However, in our case, the vertical jet consists of oil, not water, as clarified by the color image sequence in Fig. 1B. At the same time (see Fig. 1A, 11.8 ms), the water core continues its recoil phase, generating a liquid column that subsequently detaches from the substrate, resulting in one or more oil-encapsulated bouncing water drops.

To understand the conditions under which core rebound occurs, we performed a systematic study of various impact conditions, using the water volume fraction, α, and the drop impact height *h* as the main parameters. *h* affects not only the impact speed and thus the Weber number (*h* approximately ∝ *V*^2^ ∝ *We*), as for the case of single-phase drops, but also the relative position of the core within the oil shell, as explained below.

**Fig. 2:**
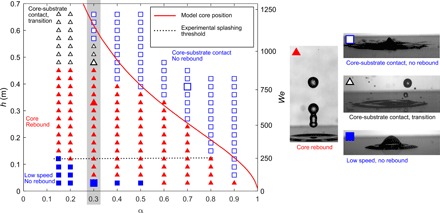
Map of the rebound behavior after impact on a hydrophilic surface as a function of the water volume fraction α and the impact height *h* (left axis) and Weber number *We* (right axis), for compound drops produced with the coaxial needle method. Closed squares, no rebound; closed triangles, rebound (core-shell rebound); open triangles, core-substrate contact, transition zone; open squares, core-substrate contact, no rebound. Shaded area: This region (α = 0.3) refers to the investigation detailed in Figs. 3 and 4. Magnified symbols correspond to the images on the right. The solid line indicates the height from which the water core can sink to the bottom of the drop, obtained by numerical integration of Eqs. 1 and 2. The *We* axis corresponds to α = 0.3 but is representative for all α since the dependence on α is small.

A map of the different impact outcomes is shown in Fig. 2. Four different impact outcomes are highlighted in the map: (i) core rebound (red triangles), (ii) low-speed drop deposition, with no rebound (blue closed squares), (iii) no rebound at high speed (blue open squares), and (iv) a transition regime (black open triangles), in which the rebounded volume is substantially lower than in regime (i).

The results make clear that there is both a lower and an upper limit for core rebound. These limits are associated with two different mechanisms. For the upper limit, the transition from core rebound to no rebound is sharp for α > 0.3, i.e., the rebounding volume drops to zero when Weber number reaches a threshold value. Differently, for α ≤ 0.3, there is a transition regime. In this regime, the bouncing volume first reduces to a substantially lower but nonzero value before it eventually goes to zero, as will be detailed below.

For completeness, the limit for splashing is indicated by a dotted line and is constant for water volume fractions up to α ≈ 0.8. This means that the presence of water does not influence oil splashing at the contact line. Previous studies (*18*) have shown that the presence of water affects oil splashing and, thus, the corresponding threshold but only at the highest water volume fraction, where α → 1.

The lower and upper limits for core rebound are due to two different mechanisms. The lower rebound limit can be explained by the energy input that is required to overcome surface energy to separate the core from the outer oil shell. In the Supplementary Materials, we show that this yields a minimum *We* for rebound *We*_min_ ∝ α^−1/3^, which correctly predicts a decreasing trend with α. However, a quantitatively accurate model should include other effects, such as viscous dissipation, that were not considered to derive the scaling law.

The upper rebound limit is related to the instability of the lubricating oil layer. In the next sections, we demonstrate the occurrence of this instability and derive a model to predict the impact height above which this occurs. The numerical solution of this model, based on Eqs. 1 and 2, which are derived and presented below, yields the solid line in Fig. 2.

### Rupture of the lubricating oil layer

**Fig. 3:**
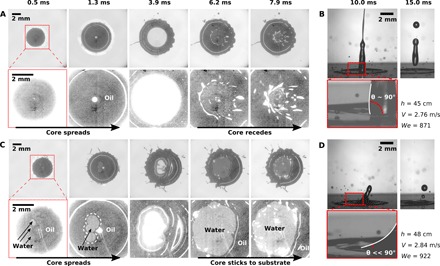
Core-substrate contact. Impact of coaxially produced drops (α = 0.3) on a hydrophilic surface. (**A** and **B**) For *h* = 45 cm (*We* = 871), the water core spreads on top of the lubricating oil layer without wetting the glass and recoils smoothly (A, bottom view reflection), resulting in core rebound (B, side view). (**C** and **D**) For *h* = 48 cm (*We* = 922), the lubricating oil layer ruptures, causing the water core to stick to the hydrophilic surface (C), suppressing the recoil, and resulting in a strongly reduced rebounded volume (D). In the bottom view reflection images (A and C), oil-substrate contact is recognizable as darker areas and water-substrate contact as lighter areas. The contrast of the magnified images in the bottom rows of (A) and (C) was enhanced during postprocessing. Overexposed areas in these images correspond to secondary reflections due to the liquid-air interface being parallel to the substrate, as is clearly visible at 3.9 ms, when the drop reaches maximum spreading.

For sufficiently high impact speed, the oil layer becomes unstable. This leads to an irreversible wetting of the glass substrate by water, causing the water core to stick to the surface. When this happens, core rebound is either strongly (for α ≤ 0.3) or completely (for α > 0.3) suppressed, as illustrated in Fig. 3.

To demonstrate the rupture of the lubricating oil layer and its correlation with rebound suppression, we took advantage of complementary bottom and side view imaging of the impact event (see setup schematic in the Supplementary Materials). In particular, for the bottom view imaging, we exploited the difference in reflectance of media with different refractive indices. By shining a beam of light on the bottom of the glass slide, close to normal incidence, the beam is subsequently reflected toward the camera. The recorded intensity on the camera is therefore a function of the refractive index of the medium touching the glass surface.

Figure 3 shows impact of compound drops (α = 0.3) on hydrophilic surfaces from impact heights *h* = 45 cm (*We* = 871) and *h* = 48 cm (*We* = 922). As detailed in the Supplementary Materials, water-substrate contact is recognizable as brighter areas and oil-substrate contact as darker areas. Figure 3C shows that the rupture of the lubricating oil film occurs during the early impact dynamics, establishing core-substrate contact. The core-substrate contact area starts out as a few small patches, similar to the breakup of an air film below an impacting drop on a dry solid surface (*40*–*42*). These patches rapidly expand radially outward. The strong hydrophilicity of the surface prevents the water-oil-substrate contact line from moving inward again, resulting in a reduced or completely absent rebound of the core.

The side view images also show indirect evidence of core-substrate contact, through the dynamic contact angle evolution at the water-oil contact line. In the case of a bouncing drop, water recoils on top of the lubricated surface, with a dynamic contact angle slightly above 90°, as typically observed on hydrophobic surfaces (*43*, *44*) and on liquid infused surfaces (*45*–*47*), where the viscosity of the infused layer has been shown to affect the dynamic contact angle and the contact line retraction dynamics (*46*). However, when water wets the glass substrate, the dynamic contact angle decreases substantially (≪90 ° ) (see Fig. 3D), causing water to stick to the substrate.

### Effect of substrate wetting properties

**Fig. 4:**
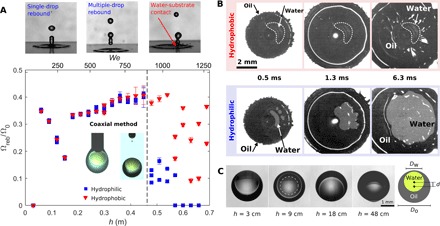
Effect of substrate wetting properties. (**A**) Total rebounded volume Ω_reb_ (water and oil) as a function of impact height *h* (approximately ∝ *We*), normalized by total impacting volume Ω_0_, for compound drops produced using the coaxial needle method, with water volume fraction α = 0.3. Three regimes can be distinguished as follows: At low impact speeds, only a single drop rebounds; at intermediate impact speeds, two or more drops rebound; and at higher speeds, core-substrate contact is established, and rebound is suppressed. The core-substrate contact threshold is indicated by the dashed line. (**B**) Bottom reflection images for *h* = 54 cm on a hydrophobic (in red) and hydrophilic (in blue) substrate, showing rupture of the lubricating oil layer. On a hydrophobic substrate the core-substrate contact area remains confined, whereas on a hydrophilic substrate the core-substrate contact area rapidly expands. The image contrast was enhanced during postprocessing. (**C**) Position of the water core as a function of impact height, *h*. The water core appears larger than it is, as the oil shell acts as a magnifying lens. The actual size of the core is indicated by the dashed circle.

To clarify the effect of substrate wetting properties, we performed impact experiments on both hydrophilic glass and functionalized hydrophobic glass and compared the results. Figure 4 illustrates the main findings, including the nondimensional volume of the bouncing liquid, Ω_reb_/Ω_0_, as a function of the impact height/Weber number (see Fig. 4A) and the bottom view image sequence of the impact event (see Fig. 4B). The water volume fraction for these experiments was α = 0.3 (see the shaded area in the parameter space in Fig. 2).

Figure 4A enables a direct comparison between impacts on hydrophilic and hydrophobic surfaces and highlights the rich complexity of drop impact physics. For very low impact *We* number (*We* ≈ 50), there is no rebound, as expected, because of insufficient available energy for rebound. At intermediate speeds, for 3 cm < *h* < 48 cm (50 < *We* < 930), the core rebounds. Within this regime, the outcome is independent of the wetting properties: No difference is observed between impacts on hydrophilic and hydrophobic surfaces (see also the detailed comparison of the pinch-off height in the Supplementary Materials). At high impact velocity, for *h* > 48 cm (*We* > 930), substrate wettability effects arise: Rebound is partially or completely suppressed (with zero bouncing liquid for *We* > 1080) on a hydrophilic substrate, whereas on a hydrophobic substrate the volume of the bouncing liquid decreases but is not completely suppressed.

The role of wetting at high impact velocities becomes clear by bottom view imaging, as represented in Fig. 4B for *h* = 54 cm (*We* = 1020). On a hydrophilic surface, water touches the surface at the periphery of the impact point (already visible at 0.5 ms) as a consequence of oil layer rupture. Subsequently, the oil-water-substrate contact line rapidly moves outward, with a substantial increase in water-substrate contact area, driven by substrate hydrophilicity. On a hydrophobic substrate, the oil layer also ruptures, but the water-substrate contact area remains confined since the contact line does not move outward. Therefore, recoil and partial rebound of water from the surface is still possible, although the value of the bouncing volume is reduced and notably scattered.

Figure 4A also reveals a nonmonotonic trend for the rebounding volume in the intermediate velocity range. This trend is associated with drop fragmentation and the breakup of the elongated water column emerging in the final stages of core recoil, as previously observed in the context of single-phase drop impact on viscoelastic surfaces (*48*). As the water column forms, it can break up in one or more drops due to Plateau-Rayleigh instability (*49*). This breakup leads to propagation of capillary waves on the drop surface down to the contact line, where either positive or negative interference with contact line receding motion takes place. This interaction affects the value of the overall rebounding volume. Within the range of 50 < *We* < 310, only a single drop breaks from the water column. For 310 < *We* < 930, multiple drops are generated by the water column lift-off, and Ω_reb_/Ω_0_ is within the range of 0.3 to 0.4. If only water was bouncing, then one would expect Ω_reb_/Ω_0_ = α = 0.3. The values can be higher, because the rebounded volume consists not only primarily of water but also partly of oil, both from the emitted jet and the oil layer encapsulating the water (since σ_o_ + σ_ow_ < σ_w_). The decomposition of the total rebounded volume into separate components can be found in the Supplementary Materials.

For *We* > 930 on a hydrophobic surface, strong variations of the rebounding volume were observed. We speculate that this is due to complex interaction between capillary waves and the asymmetry of the core-substrate contact area, leading to irregular fragmentation, and thus, rebound of water.

### Effect of compound drop geometry

A relevant aspect of a compound drop generated by the coaxial method is that the position of the water core changes during the fall. This can be observed in Fig. 4C, where images of compound drops falling from different heights were captured. In case of low impact heights, the core is positioned in the upper part of the compound drop, whereas for increasing impact height (and thus falling speed), the core is positioned in the lower part of the compound drop since water has a higher density than oil. Hence, changing the drop impact height affects the impact not only trivially, because of variation of the impact Weber number, but also because of the variation of the drop geometry. In particular, the thickness of the oil layer below the water, which is crucial to promote rebound of the core, gets thinner for increasing impact heights.

**Fig. 5:**
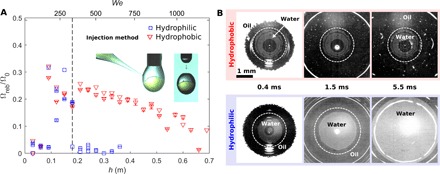
Effect of the injection method. (**A**) Total rebounded volume Ω_reb_ (water and oil) as a function of the impact height *h* (approximately ∝ *We*), normalized by the total impacting volume Ω_0_, for compound drops produced by the injection method with water volume fraction α = 0.3. The core-substrate contact threshold (dashed line) is substantially lower for drops produced by the injection method than for drops produced by the coaxial method. (**B**) Bottom view reflection images for *h* = 24 cm (*We* = 490), above the threshold. Core-substrate contact is visible. The image contrast was enhanced during postprocessing.

For direct control of the drop position, which is not possible using the coaxial needle method, we introduced a second method to produce compound drops, referred to as the injection method. This method consists of three steps as follows: (i) Oil was first infused through a single vertical needle (*D* = 1.26 mm) to produce a pendant oil drop (inset of Fig. 5A); (ii) water was subsequently injected from the side with a hydrophobized glass micropipette (*D* ~ 100 μm); and (iii) after retraction of the needle, the oil flow was restarted. The compound drop detached as soon as gravity forces overcame capillary retention forces. Using this method, water has sufficient time to sink to the bottom of the pendant oil drop, so that we can assume the oil layer thickness below the core to be similar for different impact heights. Steps (ii) and (iii) are visualized in the inset pictures in Fig. 5, which illustrates the results obtained using the injection method. Figure 5A shows the nondimensional volume of the bouncing liquid as a function of the impact height (Weber number), and Fig. 5B shows the bottom view image sequence of the impact event. To allow for a direct comparison with results from the coaxial needle method, we also performed these experiments at α = 0.3.

The results of the injection method show that rebound is strongly reduced or absent for *h* > 18 cm (*We* > 370), a much lower threshold compared to the coaxial needle method (see Fig. 4A). Therefore, there is only a small *We* range of 150 < *We* < 370 where rebound is possible on hydrophilic surfaces. This was expected since the thickness of the oil layer below the water core was smaller in the case of the injection method and, thus, the oil layer was more likely to break at lower impact speed. Moreover, it confirms the role of dynamic effects: At higher Weber number, the oil layer becomes thinner upon spreading and can break.

Nonetheless, the mechanism of oil layer rupture is the same as observed for drops generated using the coaxial needle method, as visualized by the bottom view image sequence in Fig. 5B. On the hydrophobic surface, the oil layer breaks and water wets the substrate in the early stages (as can be observed at *t* = 0.4 ms), but the water-substrate contact area does not increase further when oil spreads on the surface. Conversely, water touchdown on the hydrophilic surface is followed by an increase in the water-substrate contact area.

Above the core-substrate contact threshold for the injection method (*h* > 18 cm), the volume of core rebound on a hydrophobic substrate shows a monotonically decreasing trend. As the impact velocity increases, the amount of water sticking to the substrate increases. We observed that the injection method produces drops with a higher degree of axisymmetry than the coaxial method (compare Figs. 4B and 5B). This might explain the more regular trend observed at high impact velocities.

### Model for the core position

On the basis of the observation that the core-substrate threshold depends strongly on the position of the core, we propose to model the fall height for which the water core would reach the bottom of the compound drop, when produced with the coaxial method. We compare this height to the core-substrate threshold of rebound (Fig. 2). We consider both the outer drop and the core to be spheres with nondeformable interfaces and diameters *D*_o_ and *D*_w_, respectively (see Fig. 4C). The compound drop is assumed to be axisymmetric, but the spheres are not concentric: Relative motion of the drops is possible along the vertical axis. The center of the outer sphere has vertical coordinate *z*_o_ and the center of the inner sphere *z*_w_. We are interested in the evolution of *d* = *z*_w_ − *z*_o_ before impact.

We assume that both the core and shell start off with zero velocity and the core starts at the top of the compound drop, i.e., *d*(*t* = 0) = (*D*_o_ − *D*_w_)/2. If the compound drop were in free fall, then both the core and the shell would experience the same acceleration *g*, and relative motion would be absent, despite their density difference ρ_w_ > ρ_o_. However, the air drag on the outer drop causes a difference in acceleration, resulting in a decrease in *d*. The problem that needs to be solved is to find the theoretical height *h*_lim_ from which the core can sink from the top to the bottom of the compound drop. The water core will never truly reach the bottom during the fall since it will always be wetted by a lubricating oil layer. Therefore, we will consider the water core to be at the bottom when the lubrication force becomes dominant. We estimate the thickness of the remaining lubricating oil film to be δ_L_ ∼ 10 to 100 μm (see the Supplementary Materials). Here, we will focus on the regime where the drag force on the core can be modeled by a Stokes drag (*d* > δ_L_). Considering that δ_L_ ≪ *D*_o_, we will neglect the thickness of the lubricating layer to model the time when the water core reaches the bottom. The problem can therefore be written as *d* = −(*D*_o_ − *D*_w_)/2 for ∣*z*_o_∣ = *h*_lim_.

We assume that the compound drop experiences air drag with a constant drag coefficient, i.e., the air drag is proportional to *V*^2^, where *V* is the velocity of the compound drop. The acceleration of the compound drop can therefore be expressed as

$$\frac{dV}{dt}=-g(1-\frac{V^{2}}{V_{T}^{2}})$$ (1)

where *V* = d*z*_o_/d*t* and *V*_T_ is the terminal velocity of the compound drop, which was experimentally determined to be 5.88 m/s, as detailed in the Supplementary Materials.

To model the dynamics of the core, we consider three force components acting on the core: a gravitational component, a buoyancy component, and a drag component. We assume that the drag force on the core obeys Stoke’s law. In the Supplementary Materials, we show that the added mass only has a minor effect. Furthermore, we assume that the effect of the acceleration history (Basset force) and any internal flow inside the water core can be neglected. Under these assumptions, we derive the following equation for the time evolution of *d* (see full derivation in the Supplementary Materials)

$$\frac{dV_{\text{rel}}}{dt}=-\frac{\rho_{w}-\rho_{o}}{\rho_{w}}(g+\frac{dV}{dt})-\frac{18\mu_{o}}{\rho_{w}D_{w}^{2}}V_{\text{rel}}$$ (2)

where *V*_rel_ = d*d*/d*t* and μ_o_ is the viscosity of the oil.

Equations 1 and 2 were numerically solved to find *d*(*t*) and *z*_o_(*t*), applying the boundary conditions: *d*(0) = (*D*_o_ − *D*_w_)/2, *z*_o_(0) = 0, *V*_rel_(0) = 0, and *V*(0) = 0; *d* could be subsequently presented as a function of *z*_o_. By numerically solving *d*(*z*_o_) = −(*D*_o_ − *D*_w_)/2, the transition height *h*_lim_ was found. By repeating this procedure for different values of α (by setting *D*_w_ = *D*_o_α^1/3^), the theoretical curve *h*_lim_(α) in Fig. 2 was obtained.

The height for which the water core reaches the bottom of the compound drop is very close to the experimentally observed core-substrate contact threshold and is much higher than the threshold obtained for the injection method. This suggests that a thicker oil film below the water core is more stable during impact, leading to the rebound of the core droplet above the lower threshold. Rupture of the oil film only appears to occur when the film thickness is in a lubrication regime (*d* < δ_L_).

To verify that this observation is independent of the volume ratio, we have performed additional experiments with the injection method at different α to reproduce a similar parameter space as in Fig. 2 (shown in the Supplementary Materials). Despite the experimental limitations of the injection method on the range of α that can be produced and the oscillation effect on the initial oil film thickness, the critical core-substrate contact threshold is always substantially lower for the injection method than for the coaxial method.

When the water core reaches the bottom of the drop, the impact velocity can cause rupture of the oil film, as confirmed by the injection method experiments. This therefore explains why the critical height for core-substrate contact can be captured by this geometrical model for the coaxial method.

### Stability and rupture of the oil film

In the analysis above, we have demonstrated the importance of the oil layer thickness below the water core on the core-substrate contact threshold. However, this does not fully explain how this oil layer can remain stable under impact conditions and eventually rupture.

The stability of the oil layer below a static water drop depends on the spreading constant *S* and the van der Waals interactions (*36*, *47*, *50*). The spreading constant is defined as *S* = σ_ws_ − (σ_ow_ + σ_os_), where σ_ws_, σ_ow_, and σ_os_ are the water/solid, oil/water, and oil/solid interfacial tensions, respectively. *S* can be determined experimentally by measuring the contact angle θ*_l_* of a water drop on a glass surface immersed in oil, using the relationship *S* = −σ_ow_( cos θ*_l_* + 1) (*47*). For the hydrophilic surface, we found θ*_l_* < 40°, giving *S* < − 74 mN/m, whereas for the hydrophobic surfaces θ*_l_* = 157 ° ± 2°, giving *S* = −3.3 mN/m. Daniel *et al.* (*47*) have demonstrated that an oil layer on a glass surface below a water drop is always unstable under these conditions. The oil is completely displaced in the hydrophilic case, and it forms discrete pockets of contact in the hydrophobic case. This is consistent with our experimental observations in Fig. 4B, where the water contact area only expands on the hydrophilic substrate.

The rebound of the water core observed in our experiments presents a few analogies to the rebound of a water drop on a lubricated surface (*36*, *51*). The oil layer acts as a lubrication layer, allowing a large apparent contact angle and low contact angle hysteresis, necessary for the rebound of the water droplet. However, most of these drop impact studies use structured and hydrophobic surfaces below the lubrication layer to enhance the stability of the oil layer independently of eventual local contacts with the substrate (*47*). We also observe in our impact experiments on a flat hydrophobic surface that even above the core-substrate contact threshold, the contact regions remain limited and do not completely suppress rebound (Figs. 4 and 5). However, most film stability studies focus on gently deposited drops (*36*, *47*, *50*, *51*), and therefore, the literature and our experiments are not directly comparable.

In contrast, the water core rebound from a hydrophilic surface depends critically on the stability of the oil layer. This configuration bears more similarity to the rebound of a drop from a substrate due to the cushioning of a thin air film preventing the contact between the drop and the surface (*33*, *34*). In that configuration, rebound is observed only in a limited range of impact velocities for which the air film is stable. De Ruiter *et al.* (*33*, *34*) showed that the upper rebound limit corresponds to the minimum theoretical air thickness *h*_lim_ reaching a critical threshold *h*_c_ = 200 nm, causing the air film to rupture. The lubrication pressure of the air squeezed below the impacting drop deforms the bottom of the drop into a dimple (*52*, *53*), eventually entrapping a small air bubble when the air film ruptures (*54*). The liquid-solid contact is initiated along a ring away from the center of the impact, starting from discrete points at low impact velocities (*40*, *41*) due to the surface roughness (*42*). The rupture of the oil film in our experiments also occurs along a ring, with initial local contact points as in Figs. 3C and 4B or a complete circle as in Fig. 5B.

The stability of the oil layer in our experiments can also be explained by the lubrication pressure in the oil film below the water core. However, a direct comparison with the theoretical model used by De Ruiter *et al.* (*33*, *34*), using the oil properties for the lubrication layer, would predict a much higher limit for the core-substrate contact than what we observed experimentally (see the Supplementary Materials). It must be noted that several assumptions were made in deriving this theory, such as a high viscosity ratio (μ_o_ ∼ μ_w_, whereas for air, μ_a_ ≪ μ_w_), that are not valid in our configuration. The delayed impact of the water core on the substrate substantially dampens the impact pressure that should be considered. The higher viscosity of the cushioning layer and the surrounding fluid can also enhance the horizontal deformation of the water core when oil spreads on the solid surface, further spreading the impact pressure over a larger area. This enhanced deformation of the impacting drop was demonstrated numerically by Jian *et al.* (*55*) for a more viscous gas. The horizontal velocity of the spreading water core could also increase the thickness of the oil film entrained, as observed by Daniel *et al.* (*47*) at low capillary numbers. On the other hand, a higher shear stress on a confined oil film could further thin it (*46*) and rupture it by a shear instability (*55*).

These stabilizing mechanisms provide some hints to explain why the thicker oil layer is always stable in our experiments when the water core has not reached the bottom of the compound drop. Eventually, we expect that the critical impact velocity for core-substrate contact should depend continuously on the oil film thickness. The quantitative analysis of how this critical velocity depends on the impact conditions would require independent control of the impact velocity, the size of the water core, and the oil film thickness. A different experimental setup or numerical simulations would be needed and are left for further studies.

## DISCUSSION

Our study on rebound of compound drops consisting of immiscible liquids has revealed rich physics, extending our understanding of liquid-surface interaction acquired from classical studies of single-phase drops. Here, we have shown the existence of the self-lubrication mechanism for water-in-oil drops, which promotes water rebound even on an intrinsically hydrophilic substrate. An oil layer encapsulating a water drop acts as a lubricating layer between water and the substrate during impact, promoting rebound of water, irrespective of substrate wetting properties.

We investigated this self-lubrication mechanism to understand under which impact conditions water core rebound is achieved. Two limits were identified. At one extreme, at low impact speed, the initial kinetic energy is not sufficient to overcome surface energy and viscous losses and therefore to promote the detachment of the core from the substrate. At the other extreme, at high impact speed, the oil film becomes too thin and eventually breaks. The thickness of the oil layer depends on the drop-generation method and the impact height. Only when the oil layer is sufficiently thin, it can break. When this happens, the water contacts the substrate, and substrate wettability starts playing a role. On a hydrophilic surface, the water-substrate contact area increases, water recoil is incomplete, and as a result, rebound is partially or completely suppressed. Conversely, on a hydrophobic surface, the area wetted by water remains confined. The core recoils and rebound still occurs.

The behavior described above, and in particular, the fact that wetting properties become relevant only at high Weber number, contrasts with our intuitive understanding of the Weber number, which represents the ratio between inertial and capillary (and thus, wetting) forces. On the basis of classical drop impact studies, we are familiar with the idea that the wetting properties of the substrate should play a prominent role, especially at lower Weber numbers. However, our experiments demonstrate that only at high Weber number, wetting properties become relevant, due to the rupture of the lubricating oil layer.

The drop-generation method, a factor that has never been considered in previous studies on multiphase drop impact, also affects the impact outcome: By controlling the compound drop geometry using either the coaxial needle method or the injection method, we have demonstrated that the vertical position of the inner drop affects the thickness of the lubricating oil layer during impact and, thus, substantially affects the rebound behavior after impact. Since drop geometry plays such an important role, we highlight that future works should take particular care in evaluating and, if possible, controlling, the geometry of the compound drop before impact. In future studies, it will be valuable to investigate the effect of oil viscosity, similar to previous studies on lubricated surfaces (*46*, *47*). An increase in oil viscosity would affect both the compound drop geometry before impact (by increasing Stokes’ drag and thus affecting the mobility of the inner water drop) and the drop dynamics after impact, by increasing the stability of the oil layer under shear and decreasing the contact line retraction speed in the recoil phase, due to increased viscous dissipation.

The identification of the self-lubrication mechanism will have an impact on designing novel liquid-separation materials and devices: Self-lubrication enables dynamic separation of core and shell liquids. Moreover, our results provide useful insight for promising technologies, such as in-air microfluidics (*3*) related to coating deposition and 3D printing of complex biomaterials, cell-laden liquids for biomedical applications (*56*), and smart multicomponent materials (*13*, *57*, *58*). To mention an insight that could benefit these applications, self-lubrication allows the retraction of the core after deposition on the surface, leading to an enhanced printing resolution, provided the Weber number is sufficiently low that the core does not bounce. With respect to coating deposition, we envision that compound drops could be used to print thin films. The material that needs to be deposited could be applied as a shell encapsulating a core of immiscible liquid. The mass of the core forces the spreading of the impacting compound drop. Subsequently, self-lubrication promotes the rebound of the core, leaving behind a thin film of shell liquid on the substrate. In general, the findings of this work shed new light on the interplay between inertial and wetting phenomena on the outcome of compound drop impacts, with far-reaching implications.

## MATERIALS AND METHODS

### Chemicals and materials

Silicone oil (polydimethylsiloxane, 5 cSt) and 1*H*,1*H*,2*H*,2*H*-perfluorodecyl-triethoxysilane (97%) were purchased from Sigma-Aldrich. Isopropyl alcohol (99.7%) and acetone were purchased from Guangdong Guanghua Sci-Tech Co. Ltd. Fluorescein sodium salt was purchased from Sinopharm Chemical Reagent Co. Ltd. Water was purified with a Milli-Q system to produce type-1 water. Microscope slides were ordered from Sail Brand (catalog no.7101). Five-microliter syringes (model 1005TLL) were purchased from Hamilton Company. We fabricated glass micropipettes with a P-1000 Micropipette Puller from Sutter Instrument Company.

### Fabrication of hydrophilic and hydrophobic glass substrates

Transparent microscope slides were used to produce hydrophilic and hydrophobic substrates. The substrates were first ultrasonically cleaned, while immersing them for 10 min in isopropyl alcohol, 10 min in acetone, and 5 min in water, respectively. The slides were dried by an air gun. The microscope slides were hydrophilized by placing them in a Harrick plasma cleaner (PDC-002) for 20 min at full power.

To produce hydrophobic microscope slides, the slides were first hydrophilized, as described above, and subsequently hydrophobized by vapor deposition of 1*H*,1*H*,2*H*,2*H*-perfluorodecyl-triethoxysilane. After plasma cleaning, the slides were placed inside an airtight container, containing a cup with 0.5 ml of silane. The container was placed inside an oven at 80°C. After several minutes, the container was briefly opened to let expanded air escape. The silane solution was removed from the desiccator after approximately 10 hours. The lid was reopened, and the temperature of the oven was increased to 115°C to further promote the formation of a covalent bond between the silane and the glass surface. After 1 hour, the substrates were removed from the oven, allowing them to cool down to room temperature. A final ultrasonic cleaning step was applied to remove any traces of unbound silane.

### Production of compound drops by the coaxial method

The coaxial needle consisted of a thin 32-gauge inner needle (0.23 mm OD) inside a 21-gauge outer needle (0.81 mm OD). The flow rate through the needles was controlled by two independent syringe pumps (models: Pump 11 Pico Plus Elite and PHD 2000 Infusion, Harvard Apparatus). The volume ratio of the compound drops was controlled by varying the flow rate of the two pumps, with a combined flow rate of 10 μl/min. The compound drop detached from the coaxial needle due to its own weight. This method resulted in the production of compound drops with a fixed water volume fraction at regular time intervals. The diameter of the drops ranged from 2.3 mm (for α = 0.15) to 2.4 mm (for α = 0.9).

### Production of compound drops by the injection method

An approximately horizontal hydrophobized glass micropipette (OD ~100 μm) was inserted into an oil drop pending from a vertical 18-gauge needle tip (1.26 mm OD). Water was subsequently injected through the micropipette into the oil drop. The hydrophobic micropipette was then retracted from the drop, shedding off the water drop, which would subsequently sink to the bottom of the pendant oil drop. The flows through the vertical needle and the micropipette were independently controlled by two syringe pumps (flow rate for both pumps, 10 μl/min). Instead of varying the flow ratio of the two pumps to control α, the target volume of the water was varied. Approximately 25 s after pinch-off of the water core inside the oil drop, the infusing of oil was resumed, resulting in the pinch-off of a compound drop. By fixing the time between core pinch-off and compound drop pinch-off to approximately 25 s, a reproducible position of the water core inside the compound drop could be ensured. The compound drops produced by injection had a diameter of approximately 2.2 mm.

### Hydrophobization of needles

The coaxial needle was hydrophobized to prevent the water drop from climbing up the inner needle due to surface tension forces, obstructing the oil flow through the outer needle. Similarly, for the injection method, it is crucial that the pendant oil drop does not climb up the vertical 18-gauge needle because this results into insufficient oil volume below the vertical needle to insert the water core. The glass micropipette was also hydrophobized to ensure easy retraction from the pendant oil drop. The needles were cleaned with acetone in an ultrasonic bath for 10 min and hydrophilized by placing them in the plasma cleaner for 20 min. These hydrophilic needles were then hydrophobized by placing them in a 1% silane–isopropyl alcohol solution for 1 day.

### Drop-impact setup

The drop impacts were captured by a monochrome high-speed camera (Photron FASTCAM SA-Z) at 20,000 frames/s and a color high-speed camera (Photron FASTCAM Mini WX100) at 2000 or 3000 frames/s. We connected a Leica Z16 APO objective to the SA-Z camera, resulting in a resolution of typically 8 to 13 μm per pixel. The Mini WX100 was equipped with a ZEISS Milvus 2/50 M objective. A SUMITA LS-M352 was used as a light source. The light was guided through an optical fiber toward the setup. A diffuser was used to scatter the light. For side view imaging, the camera was placed at an angle of approximately 5°. The impact height *h* was set to zero by letting the needle tip touch the substrate surface. The impact velocity was then varied by changing the impact height from the zero. A paper tube (3 cm inner diameter) was used to prevent the effect of air flow on the falling compound drop. It was observed that airflow in the laboratory could break the axial symmetry of the compound drop.

### Determination of the rebounded volume

The rebounded volumes were obtained by processing the high-speed images digitally. A MATLAB code was implemented to detect the edges of the rebounded drops. The drops were assumed to be symmetric about the vertical axis. From the coordinates of the edge, the horizontal radius *R* was obtained as a function of the vertical coordinate *z*. By integrating π(*R*(*z*))^2^*dz* from the bottom to the top of a drop, the volume was calculated.

### Measurement of the interfacial tension with the pendant drop method

The interfacial tension between oil and water was measured by the pendant drop method. An 18-gauge needle (1.26 mm OD) was placed vertically, pointing upward in a transparent cubic container. The 5-cSt silicone oil was infused from the needle into the water pool, which results in an upside-down pendant-shape oil drop. The pendant drop images were captured by a high-speed camera (Photron FASTCAM Mini WX100) connected to a Leica Z16 APO objective. The light source was a SUMITA LS-M352. The interfacial tension was calculated following the method described in Thoroddsen *et al.* (*59*). The interfacial tension σ_ow_ of the water-oil interface was found to be 42 mN/m at 23°C, which is comparable to other silicone-oil-water systems found in literature (*60*).

## Supplementary Material

aay3499_Movie_S5.mov

aay3499_Movie_S2.mov

aay3499_Movie_S3.mov

aay3499_Movie_S8.mov

aay3499_Movie_S4.mov

aay3499_SM.pdf

aay3499_Movie_S6.mov

aay3499_Movie_S1.mov

aay3499_Movie_S7.mov

## SUPPLEMENTARY MATERIALS

Supplementary material for this article is available at http://advances.sciencemag.org/cgi/content/full/6/11/eaay3499/DC1 Section S1. Lower rebound limit Section S2. Setup for bottom view reflection contrast imaging Section S3. Original images combined bottom and side view Section S4. Acceleration and terminal velocity of the compound drop Section S5. Motion of the core with respect to the shell Section S6. Further notes on the core position model Section S7. Rebounded volume (decomposition) Section S8. Pinch-off height Section S9. Parameter space injection method Section S10. Discussion on the oil film stability and rupture Section S11. Fluid properties Section S12. Supplementary videos captions Fig. S1. Model for the lower rebound limit. Fig. S2. Setup for reflection contrast imaging. Fig. S3. Original images combined bottom and side view. Fig. S4. Impact velocity *V* as a function of impact height *h*. Fig. S5. Model for the core position with refined initial conditions. Fig. S6. Model for the core position, considering added mass. Fig. S7. Volumes of rebounded water-in-oil drops and the jet as a function of the impact height *h*, the substrate wetting properties, and the compound drop-generation method. Fig. S8. Pinch-off height *h*_po_ of the rebounding core. Fig. S9. Map of the rebound behavior after impact on a hydrophilic surface as a function of the water volume fraction α and the impact height (left axis) and Weber number *We* (right axis), for compound drops produced with the injection method. Fig. S10. Oscillations after pinch-off of a compound drop from the needle. Fig. S11. Theoretical predictions for the critical oil layer thickness. Fig. S12. Comparison of experimental data with lines of constant Weber number. Movie S1. Impact of a compound drop (α = 0.3) on a hydrophilic surface (Fig. 1A). Movie S2. Impact of a compound drop (α = 0.3) on a hydrophilic surface (color video, Fig. 1B). Movie S3. Bottom view reflection imaging of the impact of a compound drop (α = 0.3) on a hydrophilic surface (Fig. 3A). Movie S4. Bottom view reflection imaging of the impact of a compound drop (α = 0.3) on a hydrophilic surface (Fig. 3C). Movie S5. Production of a compound drop by the coaxial needle method. Movie S6. Production of a compound drop by the coaxial needle method (pinch-off). Movie S7. Production of a compound drop by the injection method. Movie S8. Production of a compound drop by the injection method (pinch-off). References (*61*–*76*)

## References

[1] Josserand C., Thoroddsen S. T., Drop impact on a solid surface. Annu. Rev. Fluid Mech. 48, 365–391 (2016).

[2] Foresti D., Kroll K. T., Amissah R., Sillani F., Homan K. A., Poulikakos D., Lewis J. A., Acoustophoretic printing. Sci. Adv. 4, eaat1659 (2018). 30182058 10.1126/sciadv.aat1659 PMC6118516

[3] Visser C. W., Kamperman T., Karbaat L. P., Lohse D., Karperien M., In-air microfluidics enables rapid fabrication of emulsions, suspensions, and 3D modular (bio)materials. Sci. Adv. 4, eaao1175 (2018). 29399628 10.1126/sciadv.aao1175 PMC5792224

[4] Prunet-Foch B., Legay F., Vignes-Adler M., Delmotte C., Impacting emulsion drop on a steel plate: Influence of the solid substrate. J. Colloid Interface Sci. 199, 151–168 (1998).

[5] Shinjo J., Xia J., Ganippa L. C., Megaritis A., Physics of puffing and microexplosion of emulsion fuel droplets. Phys. Fluids 26, 103302 (2014).

[6] Vernay C., Ramos L., Douzals J.-P., Goyal R., Castaing J.-C., Ligoure C., Drop impact experiment as a model experiment to investigate the role of oil-in-water emulsions in controlling the drop size distribution of an agricultural spray. At. Sprays 26, 827–851 (2016).

[7] Fujimoto H., Obana W., Ashida M., Hama T., Takuda H., Hydrodynamics and heat transfer characteristics of oil-in-water emulsion droplets impinging on hot stainless steel foil. Exp. Thermal Fluid Sci. 85, 201–212 (2017).

[8] V. Bertola, M. Marengo, in *Drops and Bubbles in Contact with Solid Surfaces*, M. Ferrari, L. Liggieri, R. Miller, Eds. (CRC Press, 2013), chap. 11, pp. 267–298.

[9] Laan N., de Bruin K. G., Bartolo D., Josserand C., Bonn D., Maximum diameter of impacting liquid droplets. Phys. Rev. Appl. 2, 044018 (2014).

[10] López-Herrera J. M., Popinet S., Castrejón-Pita A. A., An adaptive solver for viscoelastic incompressible two-phase problems applied to the study of the splashing of weakly viscoelastic droplets. J. Nonnewton. Fluid Mech. 264, 144–158 (2019).

[11] Vernay C., Ramos L., Ligoure C., Bursting of dilute emulsion-based liquid sheets driven by a Marangoni effect. Phys. Rev. Lett. 115, 198302 (2015). 26588421 10.1103/PhysRevLett.115.198302

[12] Zhang L., Huang J., Si T., Xu R. X., Coaxial electrospray of microparticles and nanoparticles for biomedical applications. Expert Rev. Med. Devices 9, 595–612 (2012). 23249155 10.1586/erd.12.58 PMC3618984

[13] Li X., Zhang J. M., Yi X., Huang Z., Lv P., Duan H., Multimaterial microfluidic 3D printing of textured composites with liquid inclusions. Adv. Sci. 6, 1800730 (2019). 10.1002/advs.201800730 PMC6364488 30775221

[14] Bird J. C., Dhiman R., Kwon H.-M., Varanasi K. K., Reducing the contact time of a bouncing drop. Nature 503, 385–388 (2013). 24256803 10.1038/nature12740

[15] Chen R. H., Kuo M. J., Chiu S. L., Pu J. Y., Lin T. H., Impact of a compound drop on a dry surface. J. Mech. Sci. Technol. 21, 1886–1891 (2007).

[16] Tasoglu S., Kaynak G., Szeri A. J., Demirci U., Muradoglu M., Impact of a compound droplet on a flat surface: A model for single cell epitaxy. Phys. Fluids 22, 082103 (2010). 10.1063/1.3475527 PMC2937050 20838481

[17] Gao P., Feng J. J., Spreading and breakup of a compound drop on a partially wetting substrate. J. Fluid Mech. 682, 415–433 (2011).

[18] Liu D., Tran T., Emergence of two lamellas during impact of compound droplets. Appl. Phys. Lett. 112, 203702 (2018).

[19] Liu H.-R., Zhang C.-Y., Gao P., Lu X.-Y., Ding H., On the maximal spreading of impacting compound drops. J. Fluid Mech. 854, R6 (2018).

[20] Liu D., Tran T., The ejecting lamella of impacting compound droplets. Appl. Phys. Lett. 115, 073702 (2019).

[21] Vandewalle N., Terwagne D., Gilet T., Caps H., Dorbolo S., Antibubbles, liquid onions and bouncing droplets. Colloids Surf. A 344, 42–47 (2009).

[22] Terwagne D., Gilet T., Vandewalle N., Dorbolo S., Double emulsion in a compound droplet. Colloids Surf. A 365, 178–180 (2010). 10.1021/la101096q 20491493

[23] Terwagne D., Gilet T., Vandewalle N., Dorbolo S., From a bouncing compound drop to a double emulsion. Langmuir 26, 11680–11685 (2010). 20491493 10.1021/la101096q

[24] Bremond N., Santanach-Carreras E., Chu L.-Y., Bibette J., Formation of liquid-core capsules having a thin hydrogel membrane: Liquid pearls. Soft Matter 6, 2484–2488 (2010).

[25] Ma M., Chiu A., Sahay G., Doloff J. C., Dholakia N., Thakrar R., Cohen J., Vegas A., Chen D., Bratlie K. M., Dang T., York R. L., Hollister-Lock J., Weir G. C., Anderson D. G., Core-shell hydrogel microcapsules for improved islets encapsulation. Adv. Healthc. Mater. 2, 667–672 (2013). 23208618 10.1002/adhm.201200341 PMC3814167

[26] Chiu S.-L., Lin T.-H., Experiment on the dynamics of a compound drop impinging on a hot surface. Phys. Fluids 17, 122103 (2005).

[27] Chen R.-H., Chiu S.-L., Lin T.-H., Resident time of a compound drop impinging on a hot surface. Appl. Therm. Eng. 27, 2079–2085 (2007).

[28] Tran T., Staat H. J. J., Prosperetti A., Sun C., Lohse D., Drop impact on superheated surfaces. Phys. Rev. Lett. 108, 036101 (2012). 22400761 10.1103/PhysRevLett.108.036101

[29] Quéré D., Leidenfrost dynamics. Annu. Rev. Fluid Mech. 45, 197–215 (2013).

[30] Shirota M., van Limbeek M. A. J., Sun C., Prosperetti A., Lohse D., Dynamic Leidenfrost effect: Relevant time and length scales. Phys. Rev. Lett. 116, 064501 (2016). 26918994 10.1103/PhysRevLett.116.064501

[31] Bouillant A., Mouterde T., Bourrianne P., Lagarde A., Clanet C., Quéré D., Leidenfrost wheels. Nat. Phys. 14, 1188–1192 (2018).

[32] Lyu S., Mathai V., Wang Y., Sobac B., Colinet P., Lohse D., Sun C., Final fate of a Leidenfrost droplet: Explosion or takeoff. Sci. Adv. 5, eaav8081 (2019). 31058224 10.1126/sciadv.aav8081 PMC6499590

[33] de Ruiter J., Lagraauw R., van den Ende D., Mugele F., Wettability-independent bouncing on flat surfaces mediated by thin air films. Nat. Phys. 11, 48–53 (2015).

[34] de Ruiter J., Lagraauw R., Mugele F., van den Ende D., Bouncing on thin air: How squeeze forces in the air film during non-wetting droplet bouncing lead to momentum transfer and dissipation. J. Fluid Mech. 776, 531–567 (2015).

[35] Antonini C., Bernagozzi I., Jung S., Poulikakos D., Marengo M., Water drops dancing on ice: How sublimation leads to drop rebound. Phys. Rev. Lett. 111, 014501 (2013). 23863003 10.1103/PhysRevLett.111.014501

[36] Wong T.-S., Kang S. H., Tang S. K. Y., Smythe E. J., Hatton B. D., Grinthal A., Aizenberg J., Bioinspired self-repairing slippery surfaces with pressure-stable omniphobicity. Nature 477, 443–447 (2011). 21938066 10.1038/nature10447

[37] Yuan S., Lei F., Liu Z., Tong Q., Si T., Xu R. X., Coaxial electrospray of curcumin-loaded microparticles for sustained drug release. PLoS ONE 10, e0132609 (2015). 26208167 10.1371/journal.pone.0132609 PMC4514801

[38] Mandre S., Brenner M. P., The mechanism of a splash on a dry solid surface. J. Fluid Mech. 690, 148–172 (2012).

[39] Bartolo D., Josserand C., Bonn D., Singular jets and bubbles in drop impact. Phys. Rev. Lett. 96, 124501 (2006). 16605909 10.1103/PhysRevLett.96.124501

[40] Kolinski J. M., M. Rubinstein S., Mandre S., Brenner M. P., Weitz D. A., Mahadevan L., Skating on a film of air: Drops impacting on a surface. Phys. Rev. Lett. 108, 074503 (2012). 22401209 10.1103/PhysRevLett.108.074503

[41] de Ruiter J., Oh J. M., van den Ende D., Mugele F., Dynamics of collapse of air films in drop impact. Phys. Rev. Lett. 108, 074505 (2012). 22401211 10.1103/PhysRevLett.108.074505

[42] Li E. Q., Vakarelski I. U., T. Thoroddsen S., Probing the nanoscale: The first contact of an impacting drop. J. Fluid Mech. 785, R2 (2015).

[43] Bartolo D., Josserand C., Bonn D., Retraction dynamics of aqueous drops upon impact on non-wetting surfaces. J. Fluid Mech. 545, 329–338 (2005).

[44] Antonini C., Villa F., Bernagozzi I., Amirfazli A., Marengo M., Drop rebound after impact: The role of the receding contact angle. Langmuir 29, 16045–16050 (2013). 24028086 10.1021/la4012372

[45] Chen L., Geissler A., Bonaccurso E., Zhang K., Transparent slippery surfaces made with sustainable porous cellulose lauroyl ester films. ACS Appl. Mater. Interfaces 6, 6969–6976 (2014). 24749513 10.1021/am5020343

[46] Lee C., Kim H., Nam Y., Drop impact dynamics on oil-infused nanostructured surfaces. Langmuir 30, 8400–8407 (2014). 24976266 10.1021/la501341x

[47] Daniel D., Timonen J. V. I., Li R., Velling S. J., Aizenberg J., Oleoplaning droplets on lubricated surfaces. Nat. Phys. 13, 1020–1025 (2017).

[48] Lee J. B., dos Santos S., Antonini C., Water touch-and-bounce from a soft viscoelastic substrate: Wetting, dewetting, and rebound on bitumen. Langmuir 32, 8245–8254 (2016). 27452333 10.1021/acs.langmuir.6b01796

[49] C. Tropea, A. L. Yarin, J. F. Foss, *Springer Handbook of Experimental Fluid Mechanics* (Springer Berlin Heidelberg, 2007).

[50] Carlson A., Kim P., Amberg G., Stone H. A., Short and long time drop dynamics on lubricated substrates. EPL 104, 34008 (2013).

[51] Lafuma A., Quéré D., Slippery pre-suffused surfaces. EPL 96, 56001 (2011).

[52] Mandre S., Mani M., Brenner M. P., Precursors to splashing of liquid droplets on a solid surface. Phys. Rev. Lett. 102, 134502 (2009). 19392358 10.1103/PhysRevLett.102.134502

[53] Duchemin L., Josserand C., Curvature singularity and film-skating during drop impact. Phys. Fluids 23, 091701 (2011).

[54] Thoroddsen S. T., Etoh T. G., Takehara K., Ootsuka N., Hatsuki Y., The air bubble entrapped under a drop impacting on a solid surface. J. Fluid Mech. 545, 203–212 (2005).

[55] Jian Z., Josserand C., Popinet S., Ray P., Zaleski S., Two mechanisms of droplet splashing on a solid substrate. J. Fluid Mech. 835, 1065–1086 (2018).

[56] Cole R. H., Tang S.-Y., Siltanen C. A., Shahi P., Zhang J. Q., Poust S., Gartner Z. J., Abate A. R., Printed droplet microfluidics for on demand dispensing of picoliter droplets and cells. Proc. Natl. Acad. Sci. U.S.A. 114, 8728–8733 (2017). 28760972 10.1073/pnas.1704020114 PMC5565430

[57] Truby R. L., Lewis J. A., Printing soft matter in three dimensions. Nature 540, 371–378 (2016). 27974748 10.1038/nature21003

[58] Parry E., Bolis S., Elston S. J., Castrejón-Pita A. A., Morris S. M., Drop-on-demand inkjet printing of thermally tunable liquid crystal microlenses. Adv. Eng. Mater. 20, 1700774 (2018).

[59] Thoroddsen S. T., Takehara K., Etoh T. G., The coalescence speed of a pendent and a sessile drop. J. Fluid Mech. 527, 85–114 (2005).

[60] Peters F., Arabali D., Interfacial tension between oil and water measured with a modified contour method. Colloids Surf. A 426, 1–5 (2013).

[61] Roisman I. V., Berberović E., Tropea C., Inertia dominated drop collisions. I. On the universal flow in the lamella. Phys. Fluids 21, 052103 (2009).

[62] Bereiter-Hahn J., Fox C. H., Thorell B., Quantitative reflection contrast microscopy of living cells. J. Cell Biol. 82, 767–779 (1979). 389938 10.1083/jcb.82.3.767 PMC2110483

[63] Hotta K., Sugitani A., Refractive changes in silicone oil-filled pseudophakic eyes. Retina 25, 167–170 (2005). 15689807 10.1097/00006982-200502000-00009

[64] Budwig R., Refractive index matching methods for liquid flow investigations. Exp. Fluids 17, 350–355 (1994).

[65] Thoraval M.-J., Takehara K., Etoh T. G., Thoroddsen S. T., Drop impact entrapment of bubble rings. J. Fluid Mech. 724, 234–258 (2013).

[66] Kim J.-H., Rothstein J. P., Droplet impact dynamics on lubricant-infused superhydrophobic surfaces: The role of viscosity ratio. Langmuir 32, 10166–10176 (2016). 27622306 10.1021/acs.langmuir.6b01994

[67] Kreder M. J., Alvarenga J., Kim P., Aizenberg J., Design of anti-icing surfaces: Smooth, textured or slippery? Nat. Rev. Mater. 1, 15003 (2016).

[68] Chen L., Bonaccurso E., Gambaryan-Roisman T., Starov V., Koursari N., Zhao Y., Static and dynamic wetting of soft substrates. Curr. Opin. Colloid Interface Sci. 36, 46–57 (2018).

[69] Muschi M., Brudieu B., Teisseire J., Sauret A., Drop impact dynamics on slippery liquid-infused porous surfaces: Influence of oil thickness. Soft Matter 14, 1100–1107 (2018). 29333557 10.1039/c7sm02026k

[70] Liu Y., Yan X., Wang Z., Droplet dynamics on slippery surfaces: Small droplet, big impact. Biosurf. Biotribol. 5, 35–45 (2019).

[71] Bormashenko E., Physics of pre-wetted, lubricated and impregnated surfaces: A review. Philos. Trans. R. Soc. A Math. Phys. Eng. Sci. 377, 20180264 (2019). 10.1098/rsta.2018.0264 30967071

[72] Chen L., Li Z., Bouncing droplets on nonsuperhydrophobic surfaces. Phys. Rev. E 82, 016308 (2010). 10.1103/PhysRevE.82.016308 20866726

[73] Chen L., Wu J., Li Z., Yao S., Evolution of entrapped air under bouncing droplets on viscoelastic surfaces. Colloids Surf. A 384, 726–732 (2011).

[74] Gilet T., Bush J. W. M., Droplets bouncing on a wet, inclined surface. Phys. Fluids 24, 122103 (2012).

[75] Kolinski J. M., Mahadevan L., Rubinstein S. M., Drops can bounce from perfectly hydrophilic surfaces. EPL 108, 24001 (2014).

[76] Hao C., Li J., Liu Y., Zhou X., Liu Y., Liu R., Che L., Zhou W., Sun D., Li L., Xu L., Wang Z., Superhydrophobic-like tunable droplet bouncing on slippery liquid interfaces. Nat. Commun. 6, 7986 (2015). 26250403 10.1038/ncomms8986 PMC4918357

