# Supplementary material for: Rebound of self-lubricating compound drops
Source: Sci Adv. 2020 Mar 13;6(11):eaay3499. doi: 10.1126/sciadv.aay3499 (PMC7069704; doi:10.1126/sciadv.aay3499)
Supplement: aay3499_SM.pdf [file aay3499_SM.pdf]

Science Advances
AAAS

advances.sciencemag.org/cgi/content/full/6/11/eaay3499/DC1

Supplementary Materials for

Rebound of self-lubricating compound drops

Nathan Blanken, Muhammad Saeed Saleem, Carlo Antonini*, Marie-Jean Thoraval*

*Corresponding author. Email: carlo.antonini@unimib.it (C.A.); mjthoraval@xjtu.edu.cn (M.-J.T.)

Published 13 March 2020, *Sci. Adv.* **6**, eaay3499 (2020)
DOI: 10.1126/sciadv.aay3499

The PDF file includes:

Section S1. Lower rebound limit
Section S2. Setup for bottom view reflection contrast imaging
Section S3. Original images combined bottom and side view
Section S4. Acceleration and terminal velocity of the compound drop
Section S5. Motion of the core with respect to the shell
Section S6. Further notes on the core position model
Section S7. Rebounded volume (decomposition)
Section S8. Pinch-off height
Section S9. Parameter space injection method
Section S10. Discussion on the oil film stability and rupture
Section S11. Fluid properties
Section S12. Supplementary videos captions
Fig. S1. Model for the lower rebound limit.
Fig. S2. Setup for reflection contrast imaging.
Fig. S3. Original images combined bottom and side view.
Fig. S4. Impact velocity V as a function of impact height h.
Fig. S5. Model for the core position with refined initial conditions.
Fig. S6. Model for the core position, considering added mass.
Fig. S7. Volumes of rebounded water-in-oil drops and the jet as a function of the impact height h, the substrate wetting properties, and the compound drop-generation method.
Fig. S8. Pinch-off height h_{po} of the rebounding core.
Fig. S9. Map of the rebound behavior after impact on a hydrophilic surface as a function of the water volume fraction α and the impact height (left axis) and Weber number We (right axis), for compound drops produced with the injection method.
Fig. S10. Oscillations after pinch-off of a compound drop from the needle.
Fig. S11. Theoretical predictions for the critical oil layer thickness.
Fig. S12. Comparison of experimental data with lines of constant Weber number.
Legends for movies S1 to S8
References (*61–76*)

Other Supplementary Material for this manuscript includes the following:

(available at advances.sciencemag.org/cgi/content/full/6/11/eaay3499/DC1)

Movie S1 (.mov format). Impact of a compound drop ($\alpha = 0.3$) on a hydrophilic surface (Fig. 1A).
Movie S2 (.mov format). Impact of a compound drop ($\alpha = 0.3$) on a hydrophilic surface (color video, Fig. 1B).
Movie S3 (.mov format). Bottom view reflection imaging of the impact of a compound drop ($\alpha = 0.3$) on a hydrophilic surface (Fig. 3A).
Movie S4 (.mov format). Bottom view reflection imaging of the impact of a compound drop ($\alpha = 0.3$) on a hydrophilic surface (Fig. 3C).
Movie S5 (.mov format). Production of a compound drop by the coaxial needle method.
Movie S6 (.mov format). Production of a compound drop by the coaxial needle method (pinch-off).
Movie S7 (.mov format). Production of a compound drop by the injection method.
Movie S8 (.mov format). Production of a compound drop by the injection method (pinch-off).

Section S1. Lower rebound limit

The translational kinetic energy of the core before impact can be expressed as $\frac{1}{12}\pi\rho_\mathrm{w}D_\mathrm{w}^3V^2$, where $D_\mathrm{w} = \alpha^{1/3}D_\mathrm{o}$. Just above the lower rebound limit, full rebound is observed. Therefore the surface energy of a fully rebounded core is considered, which is $\pi\sigma_\mathrm{o}D_\mathrm{w}^2$. The relevant surface tension is that of the oil, since the rebounded core is still encapsulated by a layer of oil. The interfacial energy of the water core in oil is driving the rebound from the surface. However, it is restored after rebound to the same value as before impact and therefore does not contribute to the energy balance. The thickness of this layer is neglected. Balancing the initial kinetic energy of the core with the surface energy of the rebounded core, gives $\frac{1}{12}\pi\rho_\mathrm{w}\alpha D_\mathrm{o}^3V^2 = \pi\sigma_\mathrm{o}\alpha^{2/3}D_\mathrm{o}^2$, which can be rewritten as $\rho_\mathrm{w}D_\mathrm{o}V^2/\sigma_\mathrm{o} = 12\alpha^{-1/3}$. Since $[\alpha\rho_\mathrm{w} + (1-\alpha)\rho_\mathrm{o}] \approx \rho_\mathrm{w}$, we approximate $We \approx \rho_\mathrm{w}D_\mathrm{o}V^2/\sigma_\mathrm{o}$. Therefore the minimum Weber number for core rebound is $We_\mathrm{min} \propto \alpha^{-1/3}$. This equation is illustrated in fig. **S1**. The equation correctly predicts a decrease of We_min with increasing α, though the value of the exponent does not match. Quantitatively, the observed We_min is typically an order of magnitude larger than predicted by the equation, since the equation above does not consider other relevant effects, such as viscous dissipation, which is not negligible, as well as the energy transfer between oil and water. An energy balance approach is traditionally used in the literature to understand the drop impact behaviour. We are aware of the limit of such an approach, as already highlighted by the work of Roisman and coworkers (61). Nonetheless, as a first approximation, the equation correctly describes the decreasing trend.

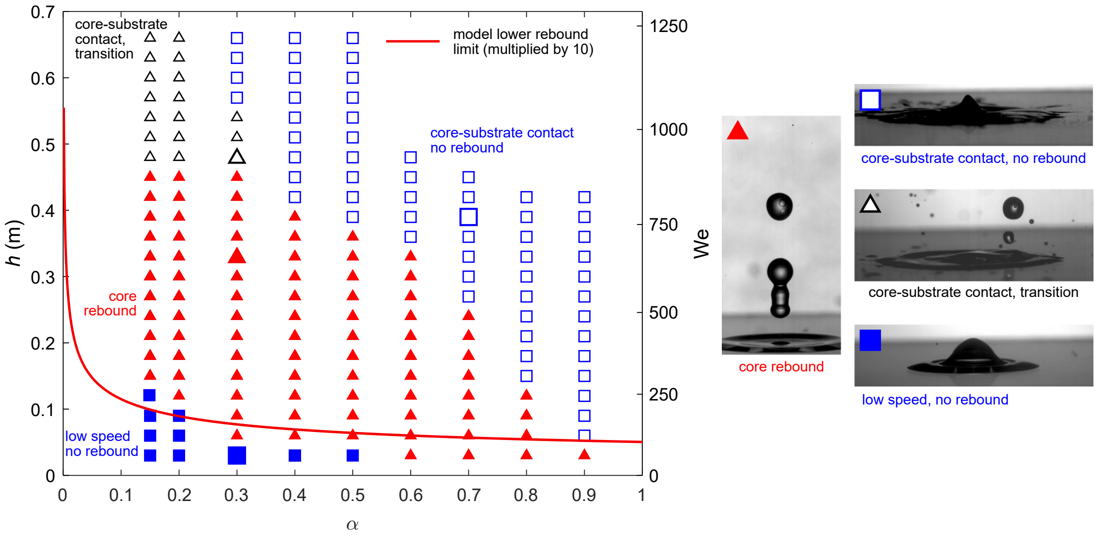

Fig. S1. Model for the lower rebound limit. The map contains the same experimental data as Fig. 2 in the main text, and includes the predicted lower rebound limit: $We_{\mathrm{min}} \propto \alpha^{-1/3}$ - see text for details.

Section S2. Setup for bottom view reflection contrast imaging

Fig. **S2** shows the setup for bottom view imaging of compound drop impact. For practical reasons, the light was directed to the glass slide via a mirror. The light is reflected from both the bottom and top surface of the glass. The light continues its path via the mirror towards the camera.

The reflectance on the interface between glass and another substance is given by Fresnel equation (62) for light at normal incidence $R = (n - n_s)^2/(n + n_s)^2$, where n_s is the refractive index of the substrate and n the refractive index of the medium on top of it. The recorded intensity on the camera sensor can be expressed as $I = R_0 I_0 + R I_0$, where I_0 is the incoming intensity, R_0 is the constant reflectance of the bottom surface of the glass slide, and R is the reflectance of the top surface of the glass slide, which depends on the medium on top of it. The different reflectance of water and silicone oil can be used to visualise the water contacting the substrate. Silicone oil has a refractive index of typically $n = 1.4 - 1.5$ $(63, 64)$, which is higher than the refractive index of water. Glass has a refractive index that is typically higher than both water and silicone oil. The refractive index of the glass is therefore closer to the index of the oil than it is to the index of water. Silicone oil will therefore appear darker than water in the images. The constant reflectance of the bottom surface of the glass results in an offset in the recorded intensity by the camera, limiting the contrast of the images. The contrast was therefore enhanced later on by digital image post-processing in MATLAB, at the expense of having a higher noise level.

In addition to the bottom interfaces of the glass, the liquid-air interface on top of the liquid also causes reflections. Because of the curved nature of this interface, those reflections are generally not directed towards the camera. However, whenever the liquid-air interface is parallel to the glass interfaces, these reflections are recorded by the camera, resulting in overexposed areas in the images. Information about the liquid-substrate interface is lost in these areas. This temporary loss of information is maximal when the drop adopts the shape of a disk at maximal spreading (see Fig. 3A in the main text at 3.9 ms).

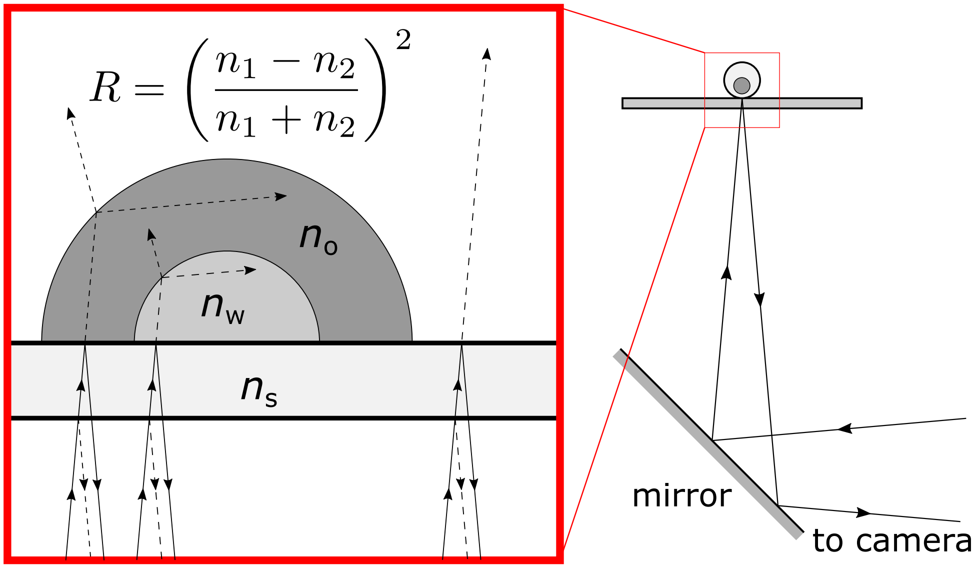

Fig. S2. Setup for reflection contrast imaging. A compound drop resides on a glass slide with refractive index n_s. Both the oil (refractive index n_o) and the water (refractive index n_w) touch the surface of the glass slide.

Section S3. Original images combined bottom and side view

Combined bottom view imaging was performed to show directly the correlation between core-substrate contact and the reduction of the rebounded volume on hydrophilic substrates. The bottom view images were taken with a monochrome Photron FASTCAM SA-Z and a Leica Z16 APO objective. The side view images were taken with a colour Photron FASTCAM Mini WX100 and a Zeiss Milvus 2/50M lens. Because of the higher frame rate and sensor sensitivity of the Photron FASTCAM SA-Z, and the larger focal depth of the Leica Z16 APO objective, the side view imaging was repeated later with this equipment. These independent side view images are shown in Fig. 3 in the main text because the results were similar. For completeness, the original side view images that were taken simultaneously with the bottom view images are shown here.

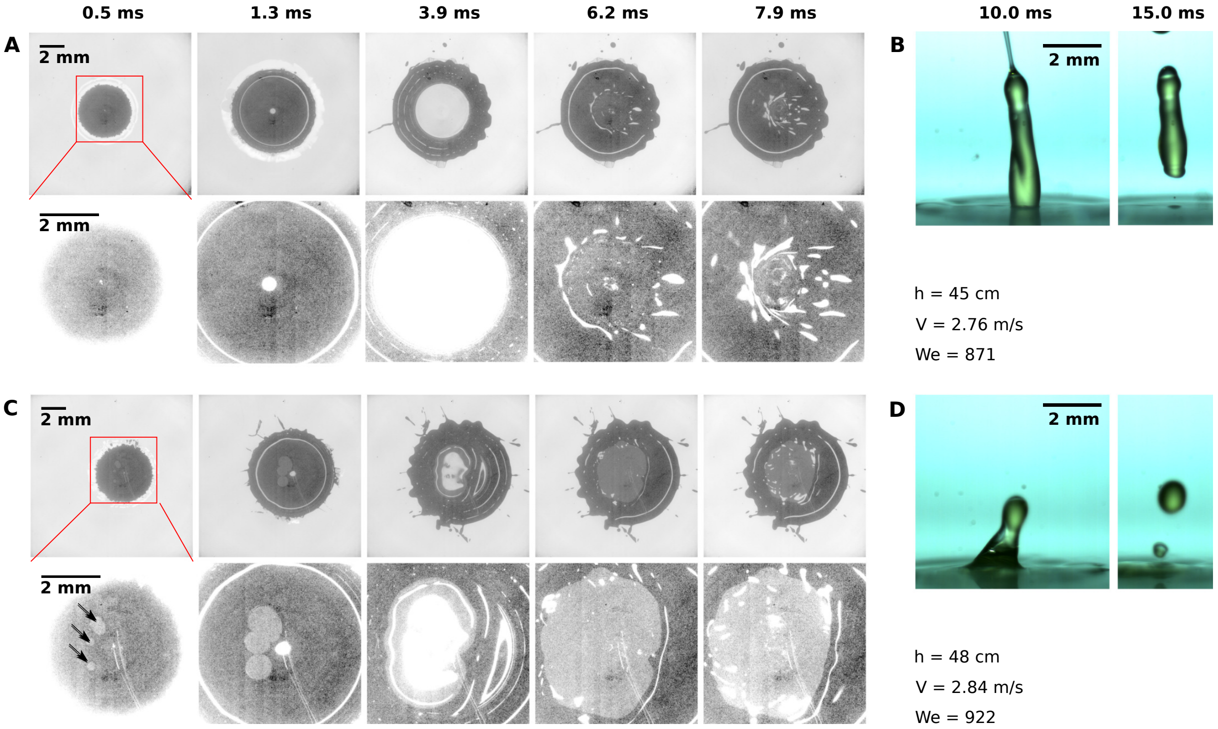

Fig. S3. Original images combined bottom and side view. Bottom view images are the same as in Fig. 3. Here, the original side view images are shown that were taken during the same compound drop impact.

Section S4. Acceleration and terminal velocity of the compound drop

The vertical coordinate of the compound drop centre of mass z_{cm} can be expressed as $z_{cm} = z_\mathrm{o} + \left[\frac{\alpha(\rho_\mathrm{w}-\rho_\mathrm{o})}{\alpha\rho_\mathrm{w}+(1-\alpha)\rho_\mathrm{o}}\right] d$. Since $\alpha(\rho_\mathrm{w} - \rho_\mathrm{o})/(\alpha\rho_\mathrm{w} + (1-\alpha)\rho_\mathrm{o}) \ll 1$, we approximate $z_{cm} \approx z_\mathrm{o}$. Therefore, we simply refer to the motion of the outer shell of the compound drop as the motion of the compound drop, and neglect the difference between z_{cm} and z_o.

The compound drop experiences two external force components: a gravitational component and air drag. We assume the drag coefficient to be constant. Thus the force on the outer drop can be expressed as

$$m_\mathrm{tot}\frac{\mathrm{d}V}{\mathrm{d}t} = -m_\mathrm{tot}g + kV^2 \tag{S1}$$

where m_tot is the total mass of the compound drop and k is some constant. It is easily shown that $k = m_\mathrm{tot}g/V_T^2$, where V_T is the terminal velocity of the drop. The acceleration of the compound drop can therefore be expressed as

$$\frac{\mathrm{d}V}{\mathrm{d}t} = -g\left(1 - \frac{V^2}{V_T^2}\right) \tag{S2}$$

V can be solved analytically as a function of the impact height h (65)

$$V = V_T\sqrt{1 - \exp\left(\frac{-2g(h - D_\mathrm{o} - h_0)}{V_T^2}\right)} \tag{S3}$$

where D_o is the diameter of the drop, and h_0, the effective pinch-off length of the drop when it separates from the nozzle. The best fit to our experimental data ($D_\mathrm{o} = 2.4$ mm) was found for a terminal velocity $V_T = 5.88$ m/s and $h_0 = 5.7$ mm. The experimental data and the best fit are shown in fig. **S4**.

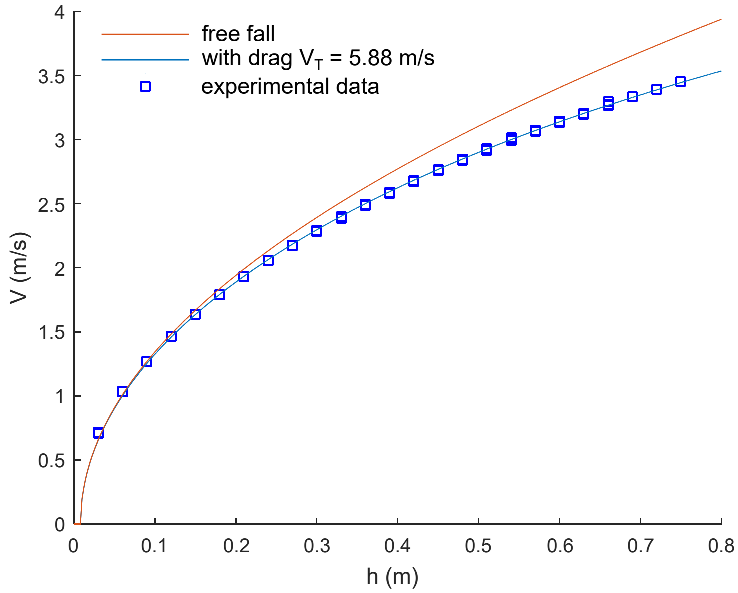

Fig. S4. Impact velocity V as a function of impact height h. The best fit to the experimental data was obtained for $V_T = 5.88$ m/s. The free fall curve $V = \sqrt{2g(h - D_o - h_0)}$ is shown for comparison.

Section S5. Motion of the core with respect to the shell

The gravitational force component acting on the core is simply given by: $F_g = -g\rho_{\mathrm{w}}\Omega_{\mathrm{w}}$, where Ω_{w} is the volume of the core. The buoyancy force for an object in an accelerating medium moving with velocity V is given by $F_b = \rho_{\mathrm{o}}\left(g + \frac{\mathrm{d}V}{\mathrm{d}t}\right)\Omega_{\mathrm{w}}$. Because of the equivalence of inertial and gravitational mass, the acceleration of the medium can simply be added to g. We assume the drag force on the core obeys Stoke's Law, i.e. $F_d = 3\pi D_{\mathrm{w}}\mu_{\mathrm{o}}V_{\mathrm{rel}}$, where μ_{o} is the viscosity of the oil and $V_{\mathrm{rel}} = V_{\mathrm{w}} - V = \frac{\mathrm{d}d}{\mathrm{d}t}$ is the velocity of the core with respect to the surrounding oil medium. After solving for V_{rel} we found that Re ~ 1 for the internal flow, justifying our approximation.

Adding up all force components and dividing through by the mass of the drop $\rho_{\mathrm{w}}\Omega_{\mathrm{w}}$, we arrive at the following equation for the velocity V_{w} of the core

$$\frac{\mathrm{d}V_{\mathrm{w}}}{\mathrm{d}t} = -g + \frac{\rho_{\mathrm{o}}}{\rho_{\mathrm{w}}}\left(g + \frac{\mathrm{d}V}{\mathrm{d}t}\right) + \frac{3\pi D_{\mathrm{w}}\mu_{\mathrm{o}}V_{\mathrm{rel}}}{\rho_{\mathrm{w}}\Omega_{\mathrm{w}}} \tag{S4}$$

Substituting $\Omega_{\mathrm{w}} = \frac{4}{3}\pi\left(D_{\mathrm{w}}/2\right)^3$ and subtracting $\frac{\mathrm{d}V}{\mathrm{d}t}$, we obtain the following equation for the relative velocity

$$\frac{\mathrm{d}V_{\mathrm{rel}}}{\mathrm{d}t} = -\frac{\rho_{\mathrm{w}} - \rho_{\mathrm{o}}}{\rho_{\mathrm{w}}}\left(g + \frac{\mathrm{d}V}{\mathrm{d}t}\right) - \frac{18\mu_{\mathrm{o}}}{\rho_{\mathrm{w}}D_{\mathrm{w}}^2}V_{\mathrm{rel}} \tag{S5}$$

Section S6. Further notes on the core position model

An improvement to the model for the core position can be achieved by applying more realistic boundary conditions than those described above. For $\alpha < 0.3$ the relative velocity of the core can be estimated when the core is approximately concentric with the shell. Under these conditions, the motion of the core can be directly observed. The theoretical transition height found with these initial conditions is given in fig. **S1**. It should also be noted that during pinch-off from the coaxial needle, the water drop is drawn at high speed into the oil shell, and is subsequently strongly decelerated. Despite strong assumptions, good agreement between the model and the experiments is observed.

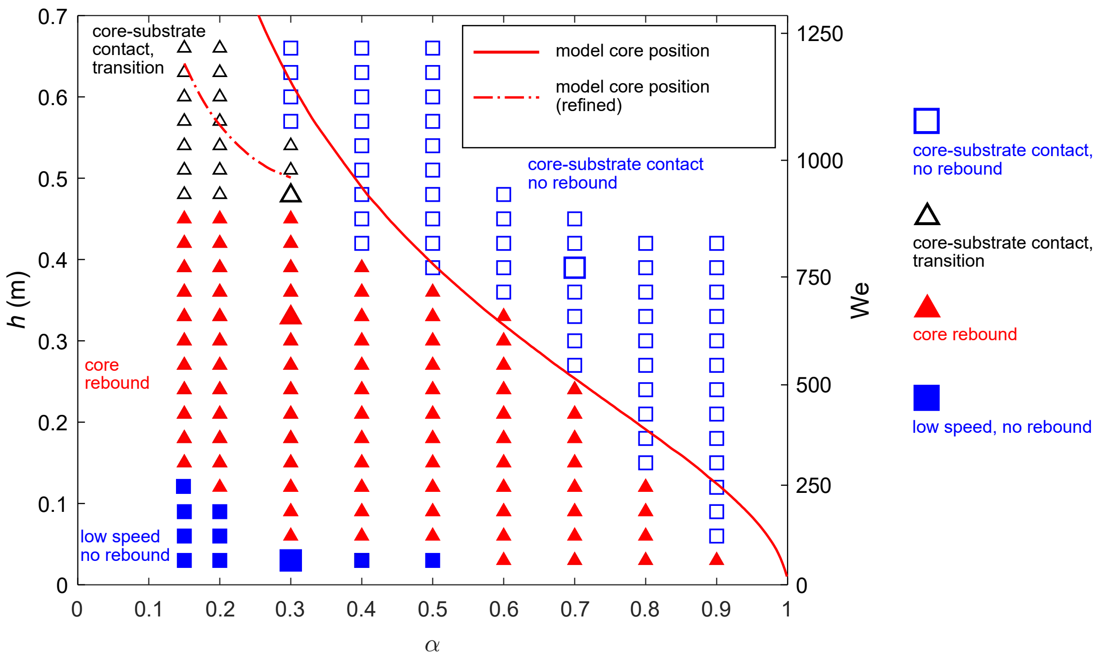

Fig. S5. Model for the core position with refined initial conditions. Solid line: as in main text. Dashed line: predicted height from which the core can sink to the bottom of the compound drop, based on refined initial conditions. The map contains the same experimental data as Fig. 2 in the main text.

In the main text we have neglected, for simplicity, the effect of the added mass on the moving core. Here we show that the effect of the added mass is minimal. As before, we consider the water core to be a non-deformable sphere, for which the added mass is $\frac{1}{2}\rho_{\text{o}}\Omega_{\text{w}}$, where Ω_{w} is the displaced volume, and ρ_{o} is the density of the surrounding liquid. Multiplying Eq. S4 by the

mass $\rho_w \Omega_w$ of the core, and adding the added mass term yields

$$\rho_w \Omega_w \frac{dV_w}{dt} = -g\rho_w\Omega_w + \left(g + \frac{dV}{dt}\right)\rho_o\Omega_w + 3\pi D_w \mu_o V_{rel} - \frac{1}{2}\rho_o\Omega_w \frac{dV_{rel}}{dt} \tag{S6}$$

Eq. S5 can now be written as

$$\frac{dV_{rel}}{dt} = -\frac{\rho_w - \rho_o}{\rho_w + \rho_o/2}\left(g + \frac{dV}{dt}\right) - \frac{18\mu_o}{(\rho_w + \rho_o/2)D_w^2}V_{rel} \tag{S7}$$

Eq. S7 was solved the same way as in the main text. The result is shown in fig. **S6**, showing that the effect of the added mass is limited.

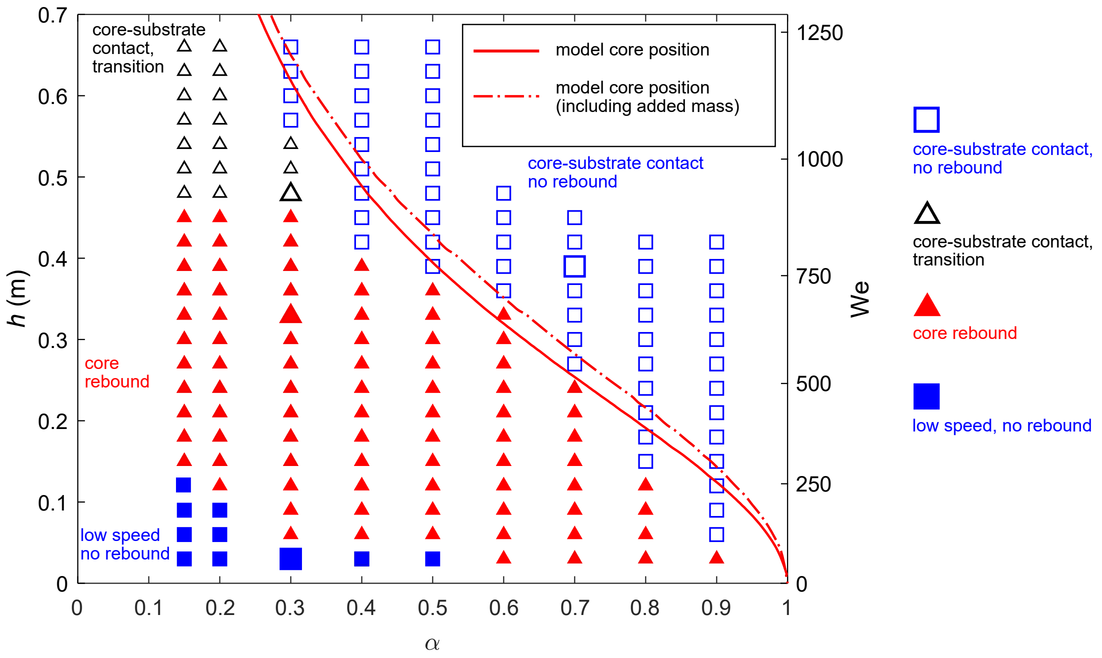

Fig. S6. Model for the core position, considering added mass. Solid line: as in main text. Dashed line: predicted height from which the core can sink to the bottom of the compound drop, taking into account the the added mass of a sphere. The map contains the same experimental data as Fig. 2 in the main text.

Section S7. Rebounded volume (decomposition)

The rebounded volumes given in Figs. 4 and 5 in the main text refer to the total rebounded volumes. In fig. **S7** we give the rebounded volumes of the individual rebounding components: the first oil-encapsulated water drop that escapes, the second oil-encapsulated water drop that escapes, and the jet. The sum of these of volumes give the total rebounded volume given in Figs. 4 and 5. The oil jet actually consists of many micrometric drops, but only the volume of the largest jet drops could be accurately determined because the smallest drops showed up pixel-sized in the images. The volumes of the larger jet drops were added up to give the jet volume.

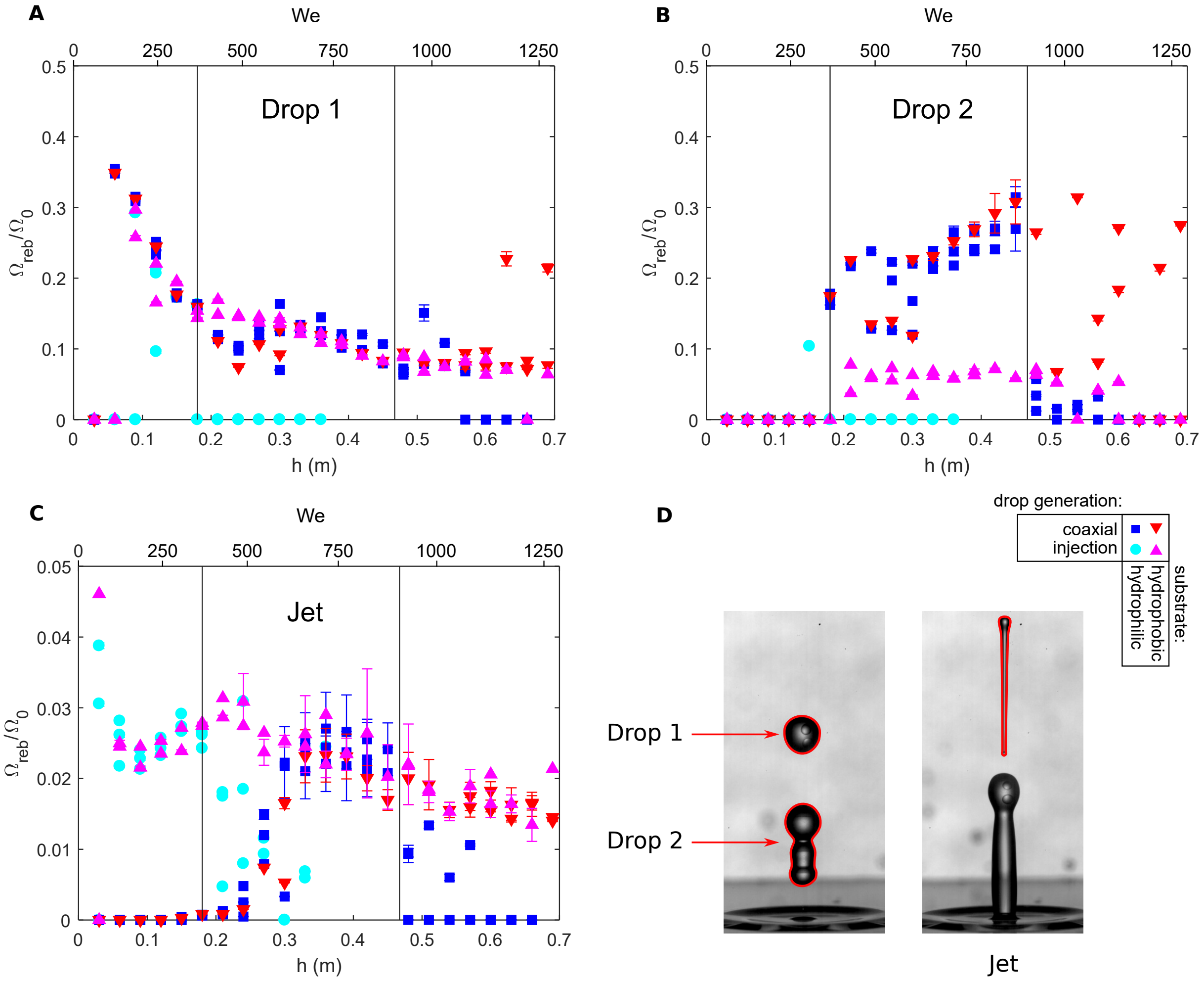

Fig. S7. Volumes of rebounded water-in-oil drops and the jet as a function of the impact height *h*, the substrate wetting properties, and the compound drop generation method. The volumes are normalised by the total impacting volume of the compound drop. The sum of these volumes is given in Figs. 4 and 5 in the main text. Note that y-axis scale for (**C**) is magnified, compared to (**A**) and (**B**). The *We*-axis corresponds to drops produced with the coaxial method, which have a slightly larger diameter than drops produced with the injection method.

Section S8. Pinch-off height

In addition to the rebounded volumes, another parameter was measured to characterise the rebound of the core. We define the pinch-off height h_{po} as the vertical distance between the pinch-off point of the first (upper) oil-encapsulated water drop that escapes. The results are given in fig. **S8**. Just like the rebounded volumes, the pinch-off heights are independent of the wetting properties of the substrate below the core-substrate contact threshold. Above this threshold, rebound is strongly or completely reduced on hydrophilic substrates.

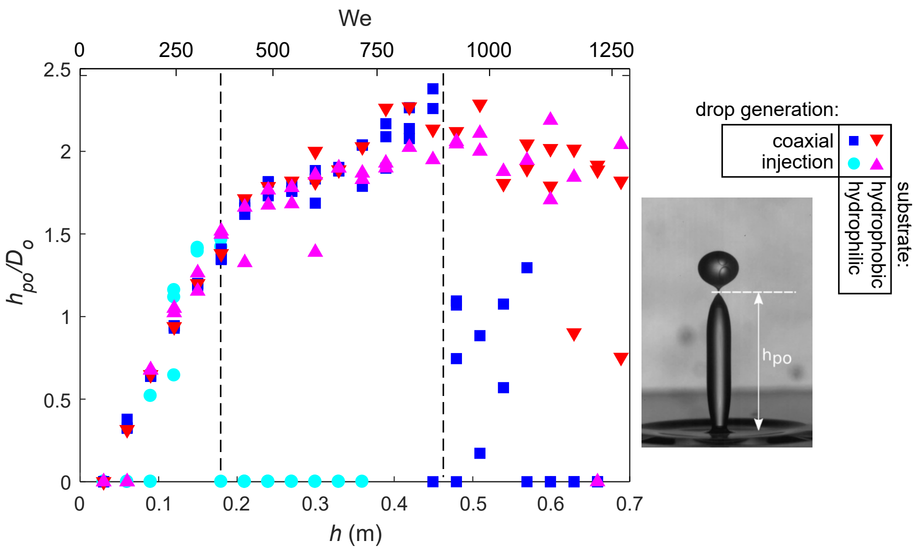

Fig. S8. Pinch-off height h_{po} of the rebounding core. In case the core splits up in two or more drops: pinch-off of the highest drop. $h_{po} = 0$ indicates no core rebound. The water volume fraction for all drops is $\alpha = 0.3$. This data was obtained from the same impact experiments as in Figs. 4 and 5 in the main text. The *We*-axis corresponds to drops produced with the coaxial method, which have a slightly larger diameter than drops produced with the injection method.

Section S9. Parameter space injection method

To gain a better understanding of the film rupture, we also produced a parameter space showing the results of compound drops produced by the injection method. The water volume ratio was limited to values up to $\alpha \approx 0.4$. Injecting a larger water drop caused the water drop to stick to the needle, and it would no longer be at the bottom of the compound drop. On the other hand, the injection method enabled drop production with smaller water volume ratios (as small as $\alpha \approx 0.05$) . The drop impact results, including also the case of $\alpha = 0$ (pure oil drop) are shown in fig. **S9**, in a similar fashion as in Fig. 2 in the main text.

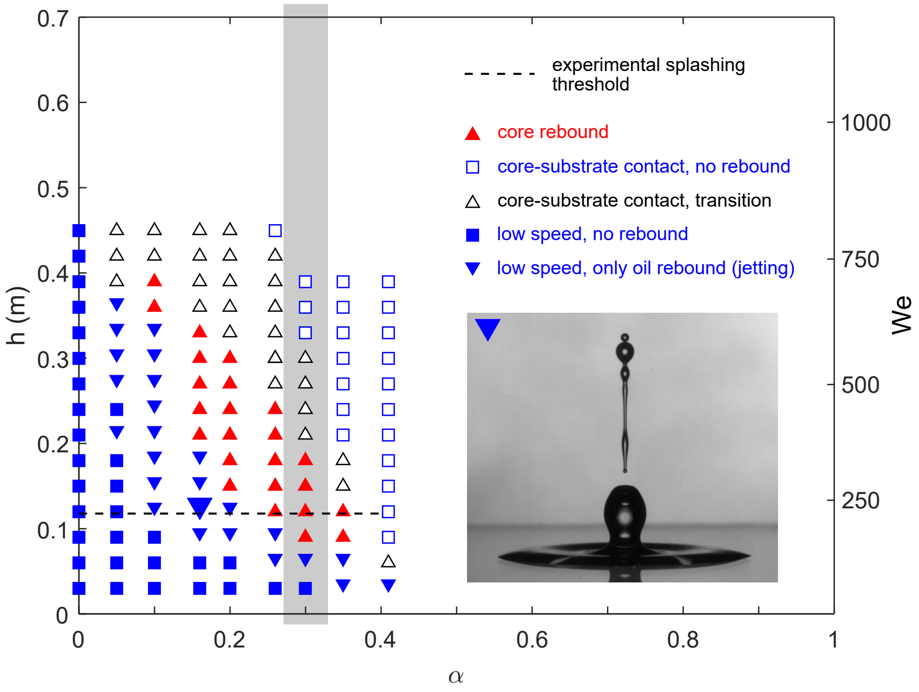

Fig. S9. Map of the rebound behaviour after impact on a hydrophilic surface as a function of the water volume fraction α and the impact height (left axis) and Weber number *We* (right axis), for compound drops produced with the injection method. Closed squares: no rebound; **closed downward triangles:** only oil rebound (jetting); **closed upward triangles:** rebound (core-shell rebound); **open triangles:** core-substrate contact, transition zone; **open squares:** core-substrate contact, no rebound; **Shaded area:** this region ($\alpha = 0.3$) refers to the investigation detailed in Fig. 5 in the main text. The magnified symbol corresponds to the image on the right. The *We*-axis corresponds to $\alpha = 0.3$, but is representative for all α since the dependence on α is small.

It must be noted that a new regime was observed with the injection method: oil jetting

was observed for heights below the lower core-rebound threshold. This behaviour was only incidentally observed with the coaxial method. Oil jetting can be attributed to the presence of a substantial volume of oil above the impacting compound drop. With the coaxial method, this volume is initially absent.

Furthermore, oscillations due to the pinch-off from the needle lift the core drop from its original position at the bottom. This is illustrated in fig. **S10**. Therefore the core may still need to undergo some gravity-driven translation (Stokes regime) to return to the bottom (lubrication regime). Because of the optical distortion due to the oil-air interface (lensing), it is challenging to quantify this oscillation-driven lifting of the core. Therefore, it is difficult to draw strong conclusions about the impact velocity to break the lubricating oil layer.

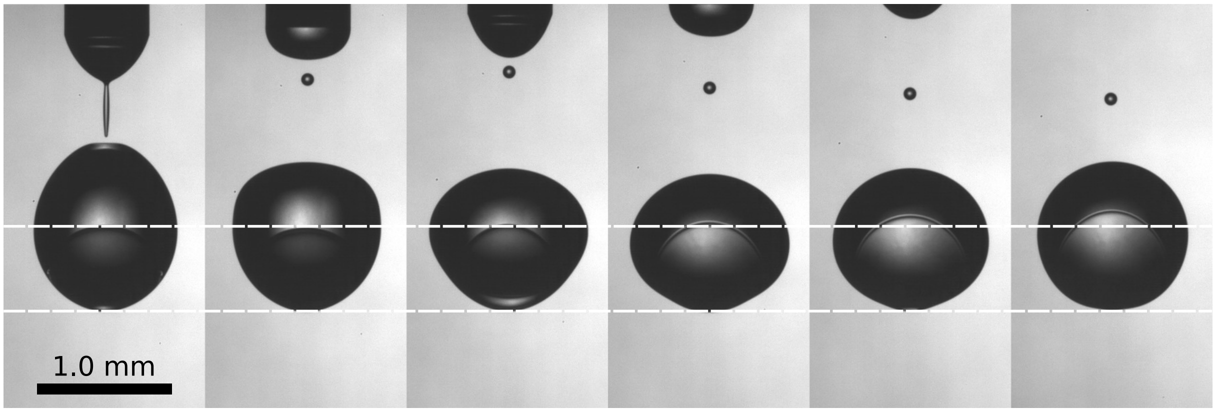

Fig. S10. Oscillations after pinch-off of a compound drop from the needle. Compound drop produced by the injection method ($\alpha = 0.1$). The time step between each image is 2 ms. Images have been vertically translated in order to align the bottoms of each drop. The upper dashed line suggests that the core drop is moving up with respect to the bottom (at least optically).

Section S10. Discussion on the oil film stability and rupture

Before compound drop impact, the water core is always wetted by the oil due to the higher surface tension of water ($\sigma_\mathrm{w} > \sigma_\mathrm{o} + \sigma_\mathrm{ow}$), as already explained in the main text. Therefore, an oil layer exists between the water droplet and the solid surface at the time of impact. We have demonstrated in the main text that the stability of this oil layer during impact plays a crucial role in the rebound of the water core on a hydrophilic surface. The transition from rebound to deposition corresponds to the impact height from which the oil layer ruptures.

We can get an order-of-magnitude estimate of the minimum oil thickness below the water core before impact using lubrication theory. Carlson *et al.* (50) give the evolution of the film thickness below the drop as $\frac{\partial \Delta}{\partial t} = \frac{\partial}{\partial x}\left(\frac{\Delta^3}{3\mu_\mathrm{o}}\frac{\partial p}{\partial x}\right)$, where Δ is the film thickness and x is the radial cylindrical coordinate. Before impact, the main pressure contribution comes from the air drag $p \sim 1/2\rho V^2$. If we assume a uniform thickness $\Delta(x) = \Delta$, and take the drop radius R as the relevant horizontal length scale, we get $\frac{\partial \Delta}{\partial t} \sim \frac{\Delta^3 p}{3\mu_\mathrm{o} R^2}$. If we estimate $\frac{\partial \Delta}{\partial t}$ as $\frac{\Delta}{T}$, with T the relevant time scale, we find $\Delta \sim \sqrt{\left(\frac{3\mu_\mathrm{o} R^2}{pT}\right)}$. Taking the fall time $\sim \sqrt{2h/g}$ as T, we find a thickness of order 10 – 100 μm.

We have discussed in the manuscript the rebound observed here to the rebound of a water drop on a lubricated surface (36, 45, 46, 51, 66–71) or due to air cushioning (33, 34, 72–76). We can compare our maximum impact velocity threshold for oil film rupture to the critical impact velocity criterium for air film rupture observed by De Ruiter *et al.* (2015) (33, 34). They have shown that the air film ruptures if the minimum film thickness h_{min}, predicted by Mandre, Mani & Brenner (2009) (52), becomes smaller than a critical thickness $h_c = 200$ nm. They give the minimum thickness as (33)

$$h_{min} = 5R\, Oh_g^{8/9}\, We^{-10/9}$$
$$Oh_g = \frac{\mu_\mathrm{g}}{\sqrt{\sigma\rho R}}$$
$$We = \frac{\rho R V^2}{\sigma}$$

This can be reformulated as a critical impact velocity V_c

$$V_c^2 = \left(\frac{5}{h_c}\right)^{9/10}\left(\frac{\mu_g^4\sigma^3}{\rho^7}\right)^{1/5} R^{-1/2} \tag{S8}$$

Replacing the gas viscosity μ_g in the equation by the oil viscosity μ_o and using the oil-water interfacial tension σ_ow and the water droplet size R_w, gives the resulting threshold shown in

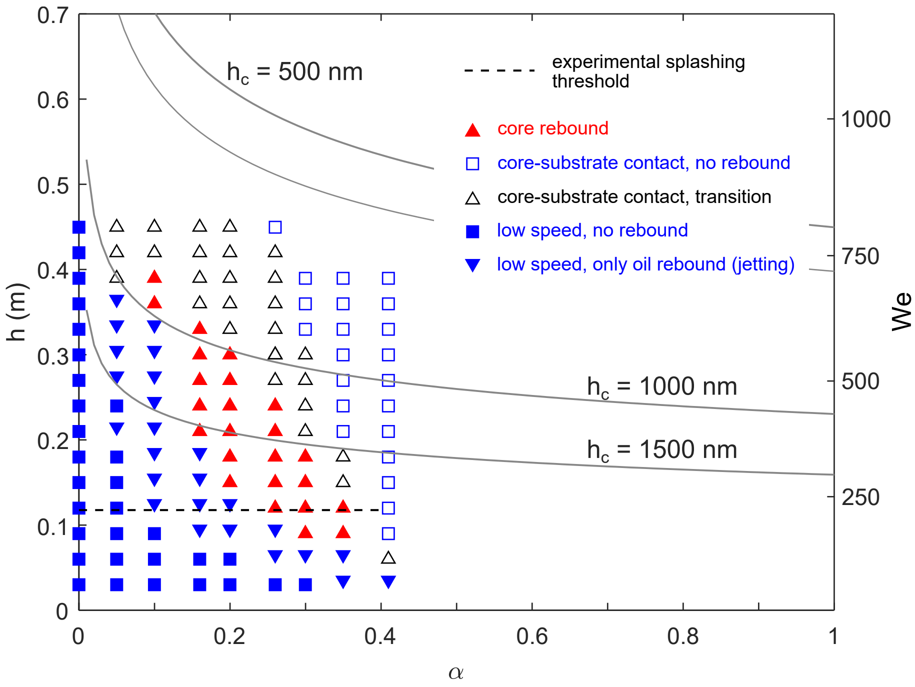

Fig. S11. Theoretical predictions for the critical oil layer thickness. Same data as in fig. S9. The solid lines correspond to the break-up limit predicted by the theory in Eq. S8 for different values of the critical film thickness h_c (33, 34, 52).

fig. **S11** for several values of h_c. Although the theory predicts a decreasing trend, as observed in our experiments, the predicted threshold is much higher and decreases more slowly, suggesting a different rupture mechanism than for the air film. In the limit of $R_w = R_o$, the theoretically predicted impact height (using $h_c = 200$ nm) would be 125 cm ($V = 4.2$ m/s, $We = 1950$), which is much higher than the observed limit.

For comparison, fig. **S12** visualises the lines for constant Weber number, based on the water core diameter, $We_w = \rho_w D_w V^2/\sigma_{ow}$. Although a decreasing trend can be observed, the transition does not occur at a constant Weber number.

It must be noted that several assumptions were made in deriving this theory, such as a high viscosity ratio ($\mu_o \sim \mu_w$, whereas for air $\mu_a \ll \mu_w$), that are not valid in our configuration. The finite size of the drop could also affect the theoretical prediction (55). Jian *et al.* (2018) (55) have demonstrated that the air film can cushion the impacting drop over a longer distance as the viscosity of the air increases, suggesting that the rupture of the air film could be due to a shear instability. The exact mechanism responsible for the oil film rupture during the impact of a compound drop therefore requires further investigation.

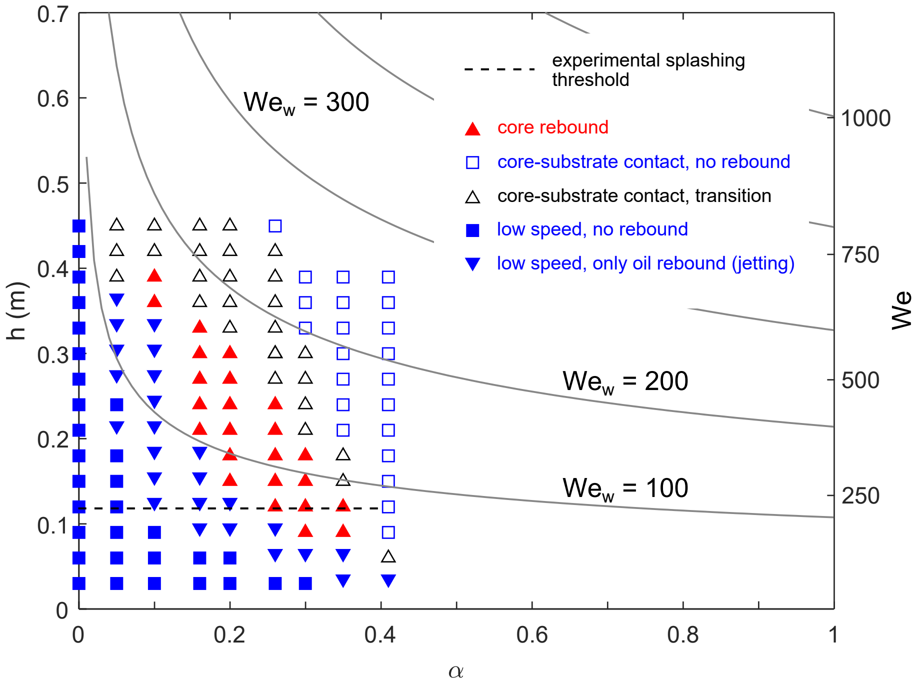

Fig. S12. Comparison of experimental data with lines of constant Weber number. Same data as in fig. S9. The solid lines are lines of constant $We_\mathrm{w} = \rho_\mathrm{w} D_\mathrm{w} V^2/\sigma_\mathrm{ow}$.

Section S11. Fluid properties

Table S1. Fluid properties at 25° C

	ρ (kg/m^3)	σ (mN/m)	μ (mPa·s)
**Silicone oil** (5 cSt)	913	19.7	4.57
**Water**	998	72.0	0.89

Section S12. Supplementary videos captions

Movie S1. Impact of a compound drop ($\alpha = 0.3$) on a hydrophilic surface (Fig. 1A). The impact height was $h = 33$ cm ($V = 2.4$ m/s, *We* $= 660$). The drop was produced by the coaxial needle method. Drop diameter $D_o = 2.4$ mm. Capture rate: 20,000 fps, playback rate: 30 fps.

Movie S2. Impact of a compound drop ($\alpha = 0.3$) on a hydrophilic surface (color video, Fig. 1B). A dye (fluorescein salt) was added to the water to distinguish it from the oil. The impact height was $h = 39$ cm ($V = 2.6$ m/s, *We* $= 770$). The drop was produced by the coaxial needle method. Drop diameter $D_o = 2.4$ mm. Capture rate: 2,000 fps, playback rate: 10 fps.

Movie S3. Bottom view reflection imaging of the impact of a compound drop ($\alpha = 0.3$) on a hydrophilic surface (Fig. 3A). The impact height was $h = 45$ cm ($V = 2.76$ m/s, *We* $= 871$). The drop ($D_o = 2.4$ mm) was produced by the coaxial needle method. Capture rate: 20,000 fps, playback rate: 30 fps. Frame dimensions: 13.3 mm $\times$ 13.3 mm.

Movie S4. Bottom view reflection imaging of the impact of a compound drop ($\alpha = 0.3$) on a hydrophilic surface (Fig. 3C). The images show the rupture of the lubricating oil layer and the wetting of the substrate by water. The impact height was $h = 48$ cm ($V = 2.84$ m/s, *We* $= 922$). The drop ($D_o = 2.4$ mm) was produced by the coaxial needle method. Capture rate: 20,000 fps, playback rate: 30 fps. Frame dimensions: 13.3 mm $\times$ 13.3 mm.

Movie S5. Production of a compound drop by the coaxial needle method. Outer needle diameter: 0.81 mm. Capture rate: 10 fps, playback rate: 30 fps.

Movie S6. Production of a compound drop by the coaxial needle method (pinch-off). Outer needle diameter: 0.81 mm. Capture rate: 3 000 fps, playback rate: 30 fps.

Movie S7. Production of a compound drop by the injection method. Outer needle diameter: 1.26 mm. Capture rate: 3 000 fps, playback rate: 30 fps.

Movie S8. Production of a compound drop by the injection method (pinch-off). Outer needle diameter: 1.26 mm. Capture rate: 5 fps, playback rate: 30 fps.
